# Reciprocal regulation between autism risk gene *POGZ* and circadian clock

**DOI:** 10.1172/jci.insight.193622

**Published:** 2026-03-17

**Authors:** Ting Wu, Jiao He, Chu-Jun Xu, Chi-Yu Li, Pingchuan Zhang, Yanfeng Wang, Shanshan Zhu, Lusi Zhang, Jingtan Zhu, Jing Zhang, Jia-Da Li, Huadie Liu

**Affiliations:** 1 Furong Laboratory & MOE Key Laboratory of Rare Pediatric Diseases, School of Life Sciences,; 2 Hunan Key Laboratory of Animal Models for Human Diseases, and; 3 Hunan Key Laboratory of Medical Genetics, Center for Medical Genetics, Central South University, Changsha, Hunan, China.; 4 Hunan Provincial University Key Laboratory of the Fundamental and Clinical Research on Neurodegenerative Diseases, Changsha Medical University, Changsha, Hunan, China.; 5 Department of Anesthesiology, Xiangya Hospital, Central South University, Changsha, Hunan, China.; 6 Department of Ophthalmology, The Second Xiangya Hospital of Central South University, Changsha, Hunan, China, Hunan Clinical Research Center of Ophthalmic Disease, Changsha, China.; 7 Hunan International Scientific and Technological Cooperation Base of Animal Models for Human Diseases, Changsha, Hunan, China.; 8 MOE Key Laboratory of Rare Pediatric Diseases, Hengyang Medical School, and; 9 Department of Cell Biology and Genetics, School of Basic Medical Sciences, Hengyang Medical School, University of South China, Hengyang, Hunan, China.

**Keywords:** Development, Genetics, Neuroscience, Behavior, Neurodevelopment

## Abstract

Sleep disturbance is a prevalent yet poorly understood comorbidity in autism spectrum disorders (ASD). Here, we uncover a bidirectional regulatory axis connecting the ASD risk gene *POGZ* to core circadian mechanisms. We demonstrate that *Pogz* is widely expressed in the suprachiasmatic nucleus (SCN), the central pacemaker of the circadian rhythms, and exhibits circadian oscillations in both the hypothalamus and liver, with its transcription directly regulated by the circadian molecule DBP through a D-box element in its proximal enhancer. *Pogz*-deficient mice exhibited prolonged circadian periodicity, impaired light-induced phase shift, delayed adaption to an 8-hour advance jet-lag, and reduced SCN c-Fos activation in response to light pulses. Mechanistically, POGZ interacts with and enhances the transcription activity of CREB, a key regulator of light-induced phase resetting. Notably, *Pogz* deletion leads to ASD-related deficits in social novelty and cognition, with cognitive impairments influenced by both photoperiod and behavioral paradigm. Our findings, thus, reveal a critical, previously unrecognized intersection between an ASD risk gene and circadian clock, offering insights into the pathogenesis of core ASD symptoms and comorbid sleep disturbances.

## Abstract 2

Study uncovers how the ASD risk gene POGZ affects circadian rhythms, reveals mechanisms underlying sleep disturbances and behavioral deficits in ASD, offering new treatment strategies.

## Introduction

Autism spectrum disorder (ASD) is a neurodevelopmental disorder characterized by social communication deficits, restricted interests, and repetitive behaviors (1). Up to 83% of individuals with ASD experience sleep disturbances, including difficulties initiating sleep, fragmented sleep, and disrupted sleep-wake cycles (2–7). These disturbances impair brain development, learning, emotional regulation, and overall quality of life (2, 3, 8–12); these impairments have been used to predict the severity of autistic symptoms (13, 14) and are known to exacerbate core ASD symptoms (15). However, their mechanistic origins in ASD remain poorly defined.

Sleep is regulated by the interplay of homeostatic and circadian processes. The circadian rhythm, which orchestrates the oscillation of behaviors, physiology, hormonal levels, and gene expression over an approximately 24-hour (24-h) cycle, is a fundamental aspect of this regulation. The suprachiasmatic nucleus (SCN), located in the hypothalamus, serves as the central pacemaker, synchronizing physiological and biochemical rhythms across the body (16). At the molecular level, circadian rhythms in nearly all cells, including SCN neurons, are maintained by transcription-translation feedback loops (TTFLs) (17), in which core proteins, such as CLOCK and BMAL1, form heterodimers that bind to E-box elements to regulate the expression of downstream genes (16, 18, 19). Additional critical *cis-*regulatory elements include the D-box and RORE, which mediate interactions with DBP/NFIL3 and REV-ERB/ROR, respectively. The D-box drives rhythmic transcription of clock-controlled genes via DBP (activator) and NFIL3 (repressor), while the RORE regulates Bmal1 expression through REV-ERB (repressor) and ROR (activator) (16, 20). The endogenous circadian rhythms do not conform precisely to 24 h; the SCN adjusts the internal clock to align with the Earth’s rotation via a process known as light entrainment (21). Light signals are transmitted by intrinsically photosensitive retinal ganglion cells (ipRGCs) to SCN neurons through the retinohypothalamic tract (RHT) (22), activating signaling pathways such as Ca^2+^, cAMP, and MAPK, which then phosphorylate CREB and upregulate immediate early genes like *Fos*, *Per1*, and *Per2* (23–25).

*POGZ* encodes a zinc finger transcription factor with an N-terminal zinc finger (ZNF) domain and a C-terminal DNA binding and transposase domain, playing a crucial role in chromatin regulation, mitotic cell cycle progression, and DNA repair (26, 27). A large-scale genetic study identified *POGZ* as a high-confidence risk gene for ASD (28). Notably, up to 75% of individuals with *POGZ* mutations exhibit sleep disturbances, including disrupted sleep-wake cycles, obstructive sleep apnea, and hypersomnolence (3, 29–35). A recent study has shown that POGZ target genes are enriched in sleep-related pathways, including circadian rhythm regulation, tau protein binding, and ATPase activator activity, further supporting a potential link between POGZ and circadian rhythms (36). However, the molecular mechanisms by which POGZ regulates circadian and sleep remain unclear (36, 37).

In this study, we uncover a reciprocal regulation between POGZ and circadian rhythms. Specifically, we demonstrate that the circadian molecule DBP regulates *Pogz* transcription by binding to a D-box element within its proximal enhancer. Conversely, POGZ directly binds to and enhances the transcriptional activity of CREB, a critical regulator of light-induced phase resetting. *Pogz-*deficient mice exhibit a significantly lengthened circadian period, impaired light-induced phase shifts, and deficits in adapting to an 8-h advance jet-lag paradigm and display ASD-like traits. Our data emphasize a connection between ASD risk genes and circadian regulation at the molecular levels, offering insights into the interplay of genetic and environmental factors that contribute to sleep disturbances in ASD.

## Results

### Circadian expression of Pogz in the SCN and liver.

In mammals, the SCN in the hypothalamus serves as the central circadian pacemaker, orchestrating rhythmic gene expression and synchronizing peripheral clocks in response to environmental light cues. The SCN comprises approximately 20,000 neurons (38), organized into 2 functionally distinct regions: the core, which contains vasoactive intestinal peptide-expressing (VIP-expressing) neurons, and the shell, primarily composed of arginine vasopressin^+^ (AVP^+^) neurons (39).

**Figure 1:**
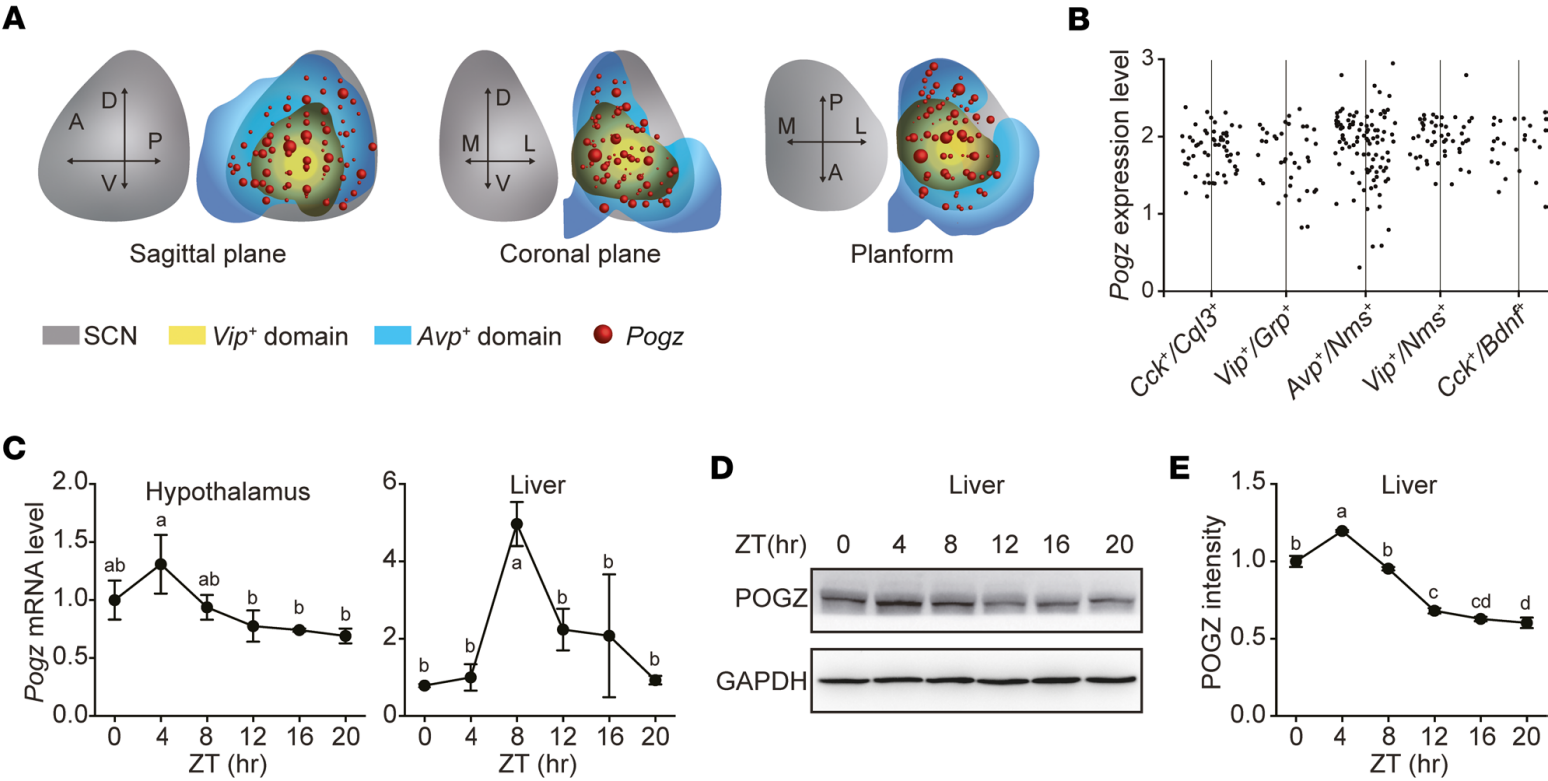
Expression and rhythmic oscillation of POGZ in the SCN and liver. (**A**) Three-dimensional spatial distribution of the *Pogz* transcripts in the suprachiasmatic nucleus (SCN) across sagittal, coronal, and axial views, based on published spatiotemporal single-cell transcriptomic data. (**B**) *Pogz* expression across 5 SCN neuronal subtypes: *Cck*^+^*/Cql3*^+^, *Vip*^+^/*Grp*^+^, *Avp*^+^/*Nms*^+^, *Vip*^+^/*Nms*^+^, and *Cck*^+^/*Bdnf*^+^. (**C**) Rhythmic oscillation of *Pogz* mRNA levels in the hypothalamus and liver under light-dark (LD) conditions. (**D**) Rhythmic oscillation of POGZ protein levels in the liver at different Zeitgeber times (ZT) under light-dark (LD) conditions, with GAPDH as the loading control. (**E**) Densitometry quantification of **D**. All data are presented as mean ± SEM (*n* = 3). Letters (a, b, c, d, ab, cd) in **C** and **E** indicate statistical differences between time points (*P* < 0.05), determined by 1-way ANOVA followed by Tukey’s post hoc test and annotated using the standard CLD method: points sharing at least 1 letter are not significantly different, whereas points with no shared letters differ significantly.

To map POGZ expression within the SCN, we analyzed a publicly available spatiotemporal single-cell transcriptomics data of the SCN (40). *Pogz* expression was detected in all 5 SCN neuronal subtypes, including *Vip^+^/Grp^+^*, *Avp^+^/Nms^+^*, *Vip^+^/Nms^+^*, *Cck^+^/Cql3^+^*, and *Cck^+^/Bdnf^+^* (Figure 1, A and B), indicating its broad involvement in SCN circuitry.

We next assessed *Pogz* mRNA levels in the hypothalamus and liver under a 12-h light–12-h dark (LD) conditions using qPCR. *Dbp*, a well-established circadian gene, was analyzed in parallel as a positive control using the same samples (Supplemental Figure 1, A and B; supplemental material available online with this article; https://doi.org/10.1172/jci.insight.193622DS1). *Pogz* exhibited circadian oscillations in both the hypothalamus and liver, with robust rhythmicity in the liver, peaking at zeitgeber time interval from 4-8 hours (ZT4–ZT8) (Figure 1C). Consistently, POGZ protein levels in the liver also oscillated, with a peak at ZT4–ZT8 (Figure 1, D and E).

### DBP positively regulates POGZ transcription.

**Figure 2:**
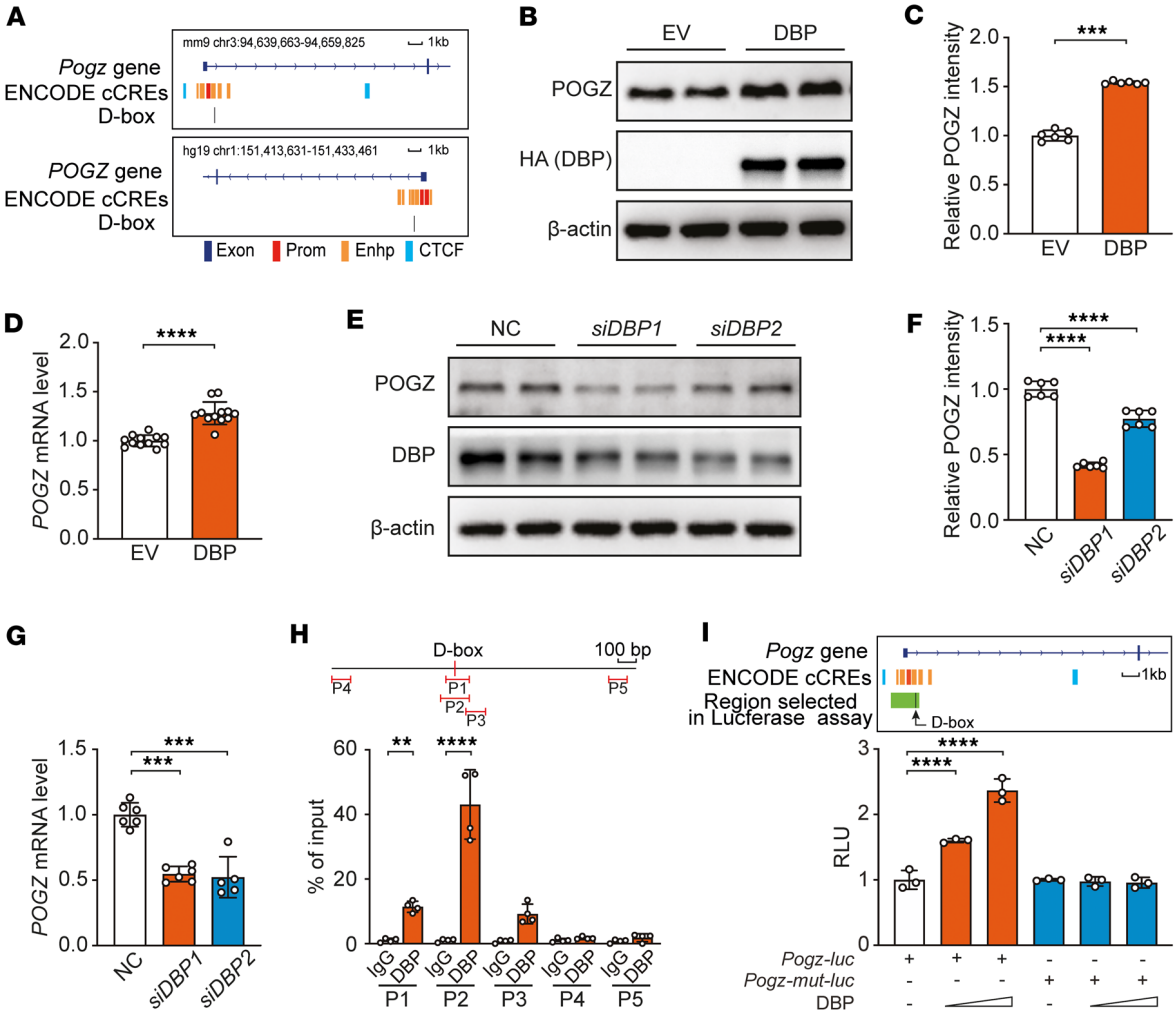
DBP regulates the expression of POGZ. (**A**) *POGZ* contains a D-box element within its proximal enhancer region in both the mouse and human genomes. (**B**) Representative immunoblot showing that overexpression of DBP (2 μg per well in a 6-well plate) in U2OS cells upregulates POGZ protein levels. (**C**) Densitometry quantification of **B** (*n* = 6). (**D**) qPCR analysis showing that DBP overexpression in U2OS cells significantly upregulates *POGZ* mRNA level (*n* = 12). (**E**) Representative immunoblot blot showing knockdown of DBP in U2OS cells reduces POGZ protein levels. (**F**) Densitometry quantification of **E** (*n* = 6). (**G**) qPCR analysis showing knockdown of DBP by siRNA (si*DBP*1, si*DBP*2) in U2OS cells (6-well plates, 5 μL of 20 μM siRNA per well) reduces *POGZ* mRNA levels (*n* = 12). (**H**) ChIP-qPCR assay demonstrating DBP binding to the D-box region of *POGZ* (*n* = 4). (**I**) Luciferase reporter assay showing that DBP enhances *Pogz* promoter activity through the D-box (*n* = 3). All data in this figure are shown as mean ± SEM. Comparisons in **C** and **D** are conducted using unpaired 1-tailed Student’s *t* test; in **F**, **G**, and **I** are conducted 1-way ANOVA; and in **H** are conducted 2-way ANOVA. ****P < 0.0001; ***P < 0.001; **P < 0.05.

Circadian transcriptional regulation involves core *cis-*regulatory elements, including the E-box (recognized by CLOCK/BMAL1), the D-box (targeted by DBP/NFIL3), and the RORE (regulated by REV-ERB/ROR) (17). Given the rhythmic expression of *Pogz*, we systematically analyzed its regulatory regions in human and mice genome for circadian-related *cis-*regulatory elements. We identified a D-box (TTATGTAA) in the first intron of the *Pogz* within a proximal enhancer region (Figure 2A), while no E-box or RORE motifs were detected.

We next assessed the functional relevance of this D-box in *Pogz* expression in U2OS cells (a widely used model for circadian studies). Overexpression of *DBP* significantly elevated the POGZ protein (Figure 2, B and C) and mRNA levels (Figure 2D), while knockdown of *DBP* led to a significant reduction of both (Figure 2, E–G).

To determine whether DBP directly binds to the D-box, we performed ChIP assays in U2OS cells transfected with pcDNA3.1-DBP-HA plasmid. DBP-bound DNA was immunoprecipitated using an HA antibody, and qPCR was performed with primers flanking the D-box. Amplicons containing or near the D-box showed significantly higher enrichment in DBP-expressing cells immunoprecipitated with HA antibody compared with the IgG control (Figure 2H).

We further confirmed that DBP regulates *Pogz* transcription via the D-box through luciferase reporter assay. *DBP* overexpression dose-dependently increased luciferase activity driven by the *Pogz* regulatory region, which includes the promoter and D-box–containing proximal enhancer (1,734 bp upstream and 270 bp downstream of the D-box motif), whereas mutation of the D-box abolished this effect (Figure 2I). Together, these results demonstrate that DBP directly bound the D-box in a proximal enhancer of *Pogz* to activate its transcription.

### Altered circadian behaviors in Pogz-deficient mice.

Homozygous *Pogz* deletions caused embryonic lethality (34, 41). To capture different aspects of POGZ in circadian regulation, we employed 3 *Pogz*-deficient mouse models: heterozygous mice (*Pogz*^+/–^) (Supplemental Figure 2, A and B) to examine dosage effects relevant to haploinsufficiency; neural-specific KO (*Pogz*^fl/fl^-*Nes*Cre [nKO]) (Supplemental Figure 2, C and D) to probe its role in the nervous system, where circadian rhythms are centrally regulated; and inducible global KO (*Pogz*^fl/fl^-UBC-CreERT2 [iKO]) (Supplemental Figure 2, C and E) to assess its requirement in adulthood while avoiding confounding developmental effects. We assessed their wheel-running activity under varying light conditions.

**Figure 3:**
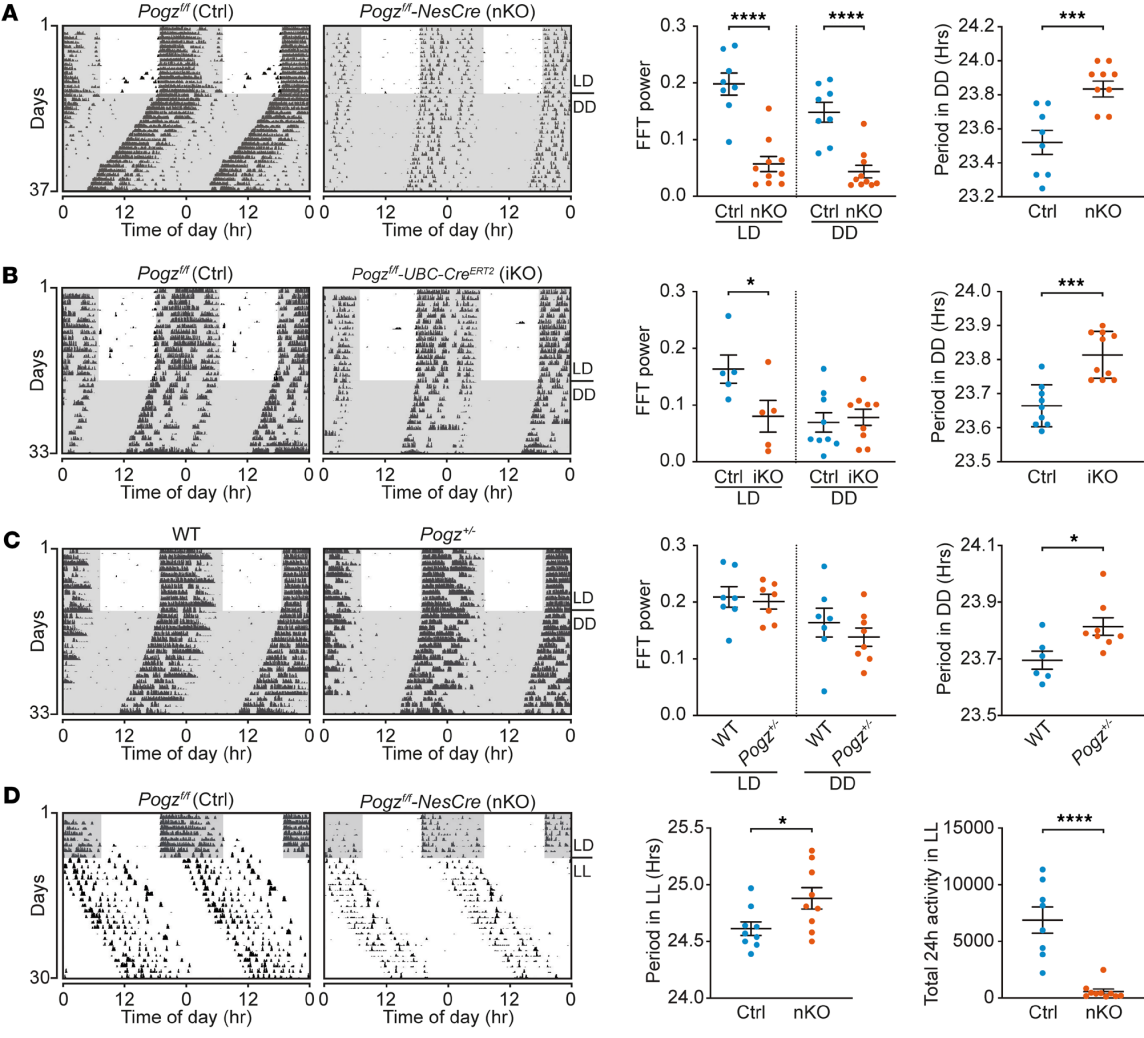
Circadian behavioral rhythms in *Pogz*-deficient mice. (**A**) Circadian locomotor activity for *Pogz^fl/fl^* (Ctrl) and *Pogz-*nKO mice under LD and DD conditions. Representative actograms (left), Fast Fourier transformation (FFT) analysis of locomotor activity rhythms (middle), and circadian period length under DD conditions (right) are included. (**B**) Circadian locomotor activity for *Pogz^fl/fl^* (Ctrl) and *Pogz*-iKO mice under LD and DD conditions. Representative actograms (left), FFT analysis of locomotor activity rhythms (middle), and circadian period length under DD conditions (right) are included. (**C**) Circadian locomotor activity for WT and *Pogz^+/–^* mice under LD and DD conditions. Representative actograms (left), FFT analysis of locomotor activity rhythms (middle), and circadian period length under DD conditions (right) are presented. (**D**) Circadian locomotor activity for *Pogz^fl/fl^* (Ctrl) and *Pogz-*nKO mice under constant light (LL) conditions. Representative actograms (left), circadian period length under LL conditions (middle), and total locomotor activity under LL conditions (right) are presented. White background indicates the lights-on condition, while the gray background indicates the lights-off condition. All data in this figure are shown as mean ± SEM (*n* = 6–10). Comparisons in all panels are conducted using unpaired 2-tailed Student’s *t* test. ****P < 0.0001; ***P < 0.001; *P < 0.05.

Under LD conditions, both control and *Pogz*-deficient mice (nKO, iKO, and *Pogz^+/–^*) exhibit clear circadian rhythms (Figure 3, A–C), with *Pogz* nKO, iKO mice displaying significantly reduced rhythmic power compared with their controls (Figure 3, A and B). These 2 groups also exhibited significantly decreased total 24-h activity (Supplemental Figure 3, A and B) and a shifted and elevated activity peak (Supplemental Figure 3, D and E), while *Pogz^+/–^* mice showed no difference from WT controls (Figure 3C and Supplemental Figure 3, C and F). No significant differences in phase angle of entrainment (the time difference between nocturnal activity onset and lights off) were observed between *Pogz*-deficient mice (*Pogz*^+/–^, iKO, and nKO) and their WT controls (Supplemental Figure 3, G–I).

Under DD conditions, all *Pogz*-deficient mice showed subtle yet significant lengthening of the free-running period. Specifically, the circadian period was prolonged by 20.4 minutes in *Pogz*-nKO mice (23.86 ± 0.04 h versus 23.52 ± 0.07 h in controls), 9.00 minutes in *Pogz* iKO mice (23.81 ± 0.02 h versus 23.66 ± 0.02 h in controls), and 6.60 minutes in *Pogz^+/–^* mice (23.81 ± 0.03 h versus 23.70 ± 0.03 h in controls) (Figure 3, A–C). Only *Pogz*-nKO mice displayed significantly reduced fast Fourier transformation (FFT) power under DD conditions (Figure 3, A–C).

Similarly, under constant light (LL) conditions, *Pogz*-nKO mice displayed a prolonged circadian period (24.88 ± 0.09 h) compared with controls (24.61 ± 0.06 h), with an increase of approximately 16.2 minutes, and showed significantly reduced 24-h total activity (Figure 3D).

The graded phenotypes (from strong deficits in nKO to moderate effects in iKO and mild effects in Het) highlight a dosage-dependent contribution of POGZ to circadian rhythm maintenance, with effects accumulating from development into adulthood. Consistently, all 3 models demonstrated that *Pogz* deficiency alters circadian behaviors in mice.

### Altered light entrainment in Pogz-deficient mice.

**Figure 4:**
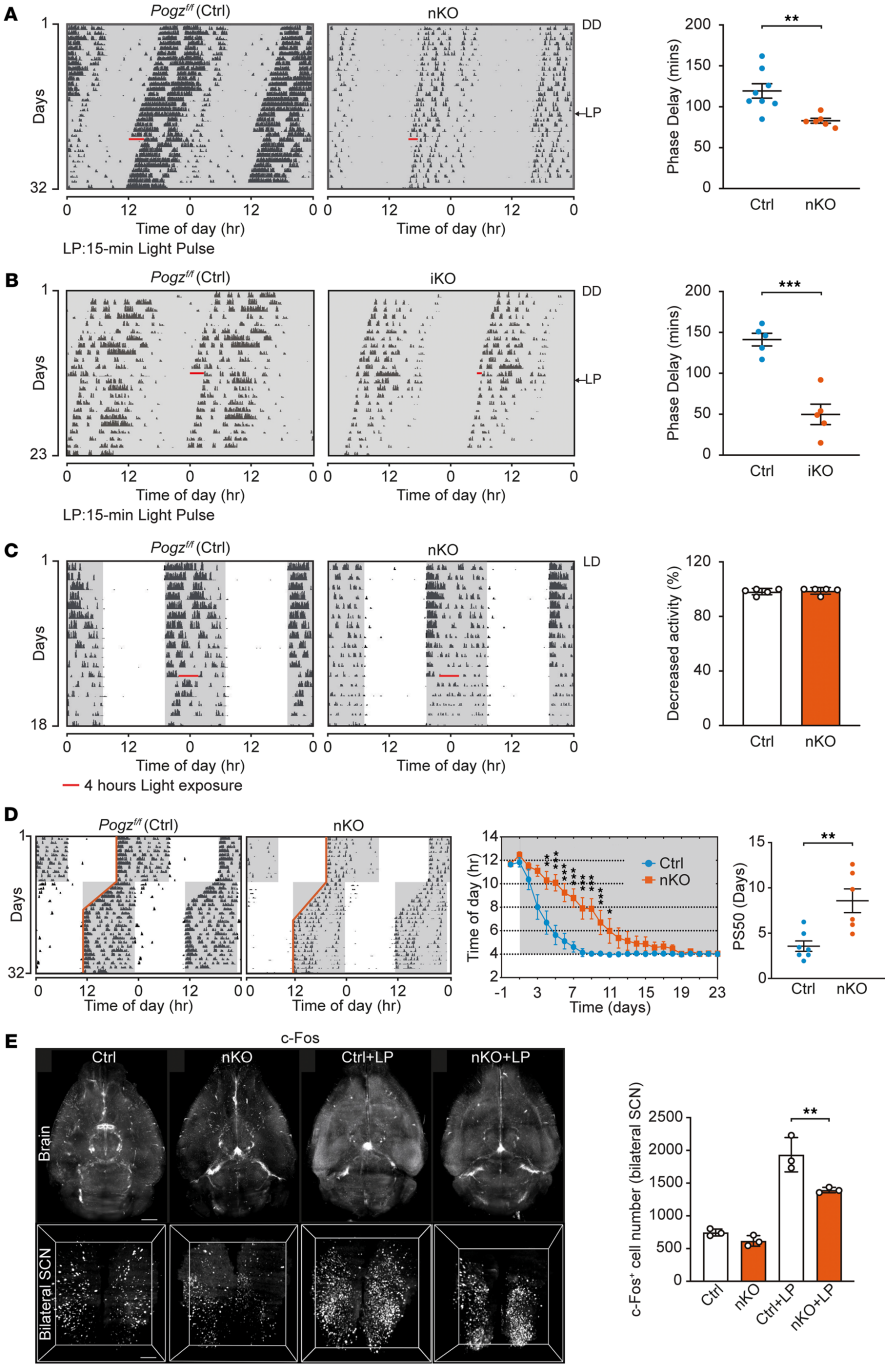
*Pogz*-deficient mice are defective in resetting the circadian clock. (**A** and **B**) Phase delays in response to a 15-minute light pulse (LP) at CT15 in *Pogz^fl/fl^* (Ctrl), *Pogz* nKO (**A**) and *Pogz* iKO (**B**). Representative actograms (left) and phase delay quantifications (Right, *n* = 5–8 per group) are presented. (**C**) Locomotor activity in *Pogz^fl/fl^* (Ctrl) and *Pogz*-nKO mice during a 4-h light exposure administered at night (ZT15–ZT19). Representative actograms (left) and quantification of light masking responses shown as the percentage reduction in activity during the 4-h light pulse compared with the same period over the previous 5 days under LD (right) are included. (**D**) Phase-shift adaptation test in a simulated jet-lag paradigm of *Pogz^fl/fl^* (Ctrl) and *Pogz*-nKO mice. Representative actograms (left), group analysis of activity onset in *Pogz^fl/fl^* (Ctrl) and *Pogz* nKO mice (*n* = 6–7 per group) (middle), and PS50 value (time to reach 50% phase shift) for *Pogz^fl/fl^* (Ctrl) and *Pogz* nKO mice (right) are included. White background indicates the lights-on condition, while the gray background indicates the lights-off condition in representative actograms of **A**–**D**. (**E**) Whole-brain c-Fos labeling and SCN quantification. Representative optical sections of 3D-rendered c-Fos^+^ cells in the whole brain and SCN from WT and *Pogz* nKO mice, under baseline or light-pulse (LP) conditions (left) and quantification of c-Fos^+^ cells in the bilateral SCN under the same conditions (right), are included (*n* = 3 for each group). All data in this figure are shown as mean ± SEM. Comparisons in **A**–**D** (right) are conducted using unpaired 2-tailed Student’s *t* test, in **D** (middle) are conducted 2-way ANOVA, and in **E** are conducted 1-way ANOVA. ***P < 0.001; **P < 0.01; *P < 0.05.

To investigate the role of POGZ in light-induced phase shift, we exposed control and *Pogz*-deficient (nKO and iKO) mice to a 15-minutes light pulse (150 lux) at early subjective night (CT15), a time point correspond to the maximal phase delay observed in mice (42) under DD condition. The light pulse at CT15 provoked a dramatic phase delay in control mice (Figure 4, A and B). However, the phase delays were significantly reduced in both *Pogz*-nKO mice (83.00 ± 3.01 min versus 119.38 ± 8.82 min in controls) and *Pogz* iKO mice (49.80 ± 12.50 min versus 141.20 ± 7.75 min in controls) (Figure 4, A and B). These results indicate that *Pogz* deficiency impairs photic entrainment.

To assess whether POGZ affects light-induced locomotor suppression (masking), we exposed mice to 150 lux light for 4 h during their active phase (ZT15-19) under an LD cycle and measured wheel-running activity. Both control and *Pogz*-nKO mice showed nearly complete suppression (*Pogz^fl/fl^*, 98.23 ± 1.04%; *Pogz* nKO, 98.79 ± 1.09%; *P* > 0.05) (Figure 4C), indicating normal light-indued locomotor suppression in *Pogz*-deficient mice.

To investigate the functional role of POGZ in circadian plasticity, we subjected *Pogz*-nKO mice and controls to an 8-h advance jet-lag paradigm. Both genotypes demonstrated progressive realignment of their locomotor activity rhythms to the shifted LD cycle yet exhibited marked differences in adaptation kinetics. Control mice achieved complete reentrainment within approximately 8 days, whereas nKO mice required around 16 days for full synchronization (Figure 4D). Quantitative analysis using the PS50 metric (time required for 50% phase correction) revealed significantly prolonged adaptation in nKO mice compared with controls (8.57 ± 1.31 days versus 3.57 ± 0.57 days), indicating compromised circadian resetting capacity (Figure 4D). As a limitation, it should be noted that LL, light-pulse, and jet-lag assays were performed only in nKO mice, which were chosen for their relatively stronger baseline circadian phenotypes.

To further probe the role of POGZ in circadian regulation at the molecular and neural circuit levels, we examined neuronal activation in response to photic input. Light-induced c-Fos expression in the SCN is a well-established marker of SCN responsiveness to environmental light cues. Using whole-brain c-Fos imaging following a light pulse, we found that baseline c-Fos expression was comparable between WT and *Pogz* nKO mice, whereas light-induced c-Fos induction in the SCN was significantly reduced in *Pogz*-nKO mice compared with WT controls (Figure 4E and Supplemental Videos 1–4), indicating that loss of POGZ blunts photic activation of the SCN.

### POGZ potentiates the transcription activity of CREB.

**Figure 5:**
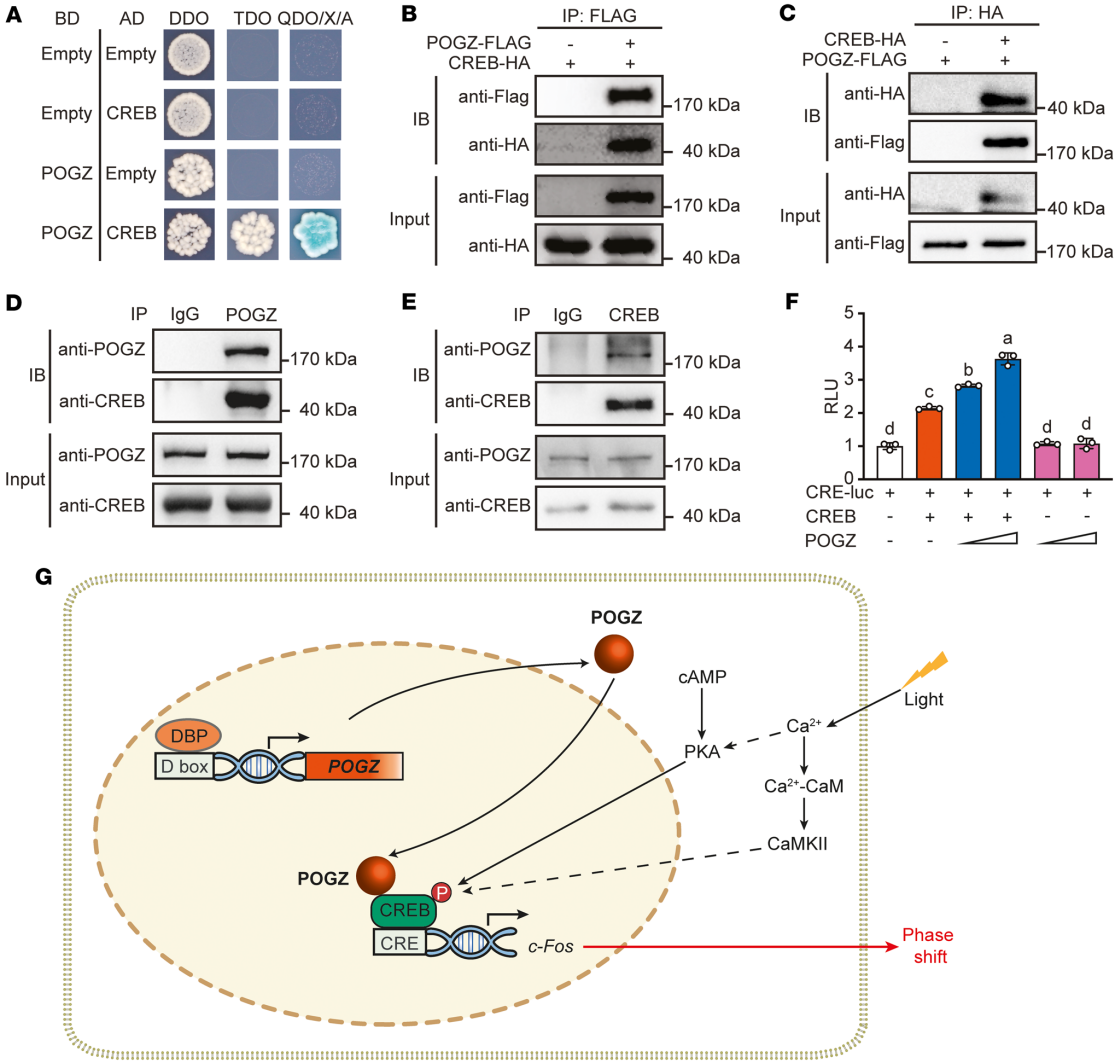
POGZ interacts with and enhances CREB-mediated transcriptional activation. (**A**) Yeast 2-hybrid assay demonstrating direct interaction between POGZ and CREB. Blue colonies on QDO/X/A (SD/-Ade/-Trp/-Leu/-His/ABA/X-α-gal) selection medium containing 10 mM 3-AT indicate a positive interaction. The experiment was repeated independently 3 times with similar results. (**B** and **C**) Co-IP assays in U2OS cells confirming the interaction between overexpressed POGZ and CREB using FLAG (**B**) or HA (**C**) antibody. (**D** and **E**) Endogenous Co-IP in mouse brain lysates confirming POGZ-CREB interaction. (**F**) Luciferase reporter assay showing that POGZ enhances CREB-mediated transcriptional activation of CRE-containing promoters. Data are presented as mean ± SEM (*n* = 3). Letters (a, b, c, d) in **F** indicate statistical differences between time points: points with different letters are significantly different (*P* < 0.05), determined by 1-way ANOVA followed by Tukey’s post hoc test and annotated using the standard CLD method. The experiment for **B**–**E** was repeated independently at least 3 times with similar results. (**G**) Schematic model summarizing the interaction between POGZ and CREB in regulating circadian genes and phase-shift adaptation.

To investigate the molecular mechanisms by which POGZ regulates circadian rhythms, we performed a yeast 2-hybrid (Y2H) screen using POGZ as bait. CREB (cAMP response element-binding protein), a key regulator of light-induced circadian entrainment (43), was identified as a POGZ-interacting protein (Figure 5A).

We confirmed the POGZ-CREB interaction via Co-IP in U2OS cells. When POGZ-Flag and CREB-HA were coexpressed, anti-Flag antibody immunoprecipitated CREB-HA and anti-HA antibody immunoprecipitated POGZ-Flag protein (Figure 5, B and C). Additionally, endogenous Co-IP assays using an anti-POGZ antibody pulled down CREB, while an anti-CREB antibody pulled down POGZ (Figure 5, D and E), confirming their interaction in a native cellular context.

To explore the functional significance of the POGZ-CREB interaction, we assessed CREB transcriptional activity using a CRE-luciferase reporter assay. Cotransfection of CRE-luciferase plasmid with *CREB* gene significantly increased luciferase activity. Notably, POGZ further enhanced CREB-mediated transcription in a dose-dependent manner, whereas POGZ alone had no effect on the CRE-luciferase activity (Figure 5F).

Taken together, our data indicate that POGZ interacts with CREB and potentiates its transcription activity, thereby facilitating light-induced phase shift (Figure 5G).

### Pogz-nKO mice exhibited ASD-related behavioral deficits.

Circadian rhythms play a crucial role in regulating mood, cognition, and social behaviors (44). POGZ is a well-established ASD risk gene (45, 46), and our data show that *Pogz*-deficient mice exhibit abnormal light entrainment, indicating impaired circadian responsiveness. Sleep and circadian disturbances are common in individuals with ASD (47). Consistently, human genetic studies link mutations in canonical clock genes to ASD (48–50), and animal models demonstrate that disruption of core clock genes leads to ASD-like social behaviors (51, 52). Based on these observations, we asked whether photoperiod influences ASD-relevant behaviors and whether POGZ mediates these effects. Accordingly, we examined social and cognitive behaviors in *Pogz*-nKO mice under 3 photoperiods: regular (12L:12D), short (8L:16D), and long (16L:8D).

**Figure 6:**
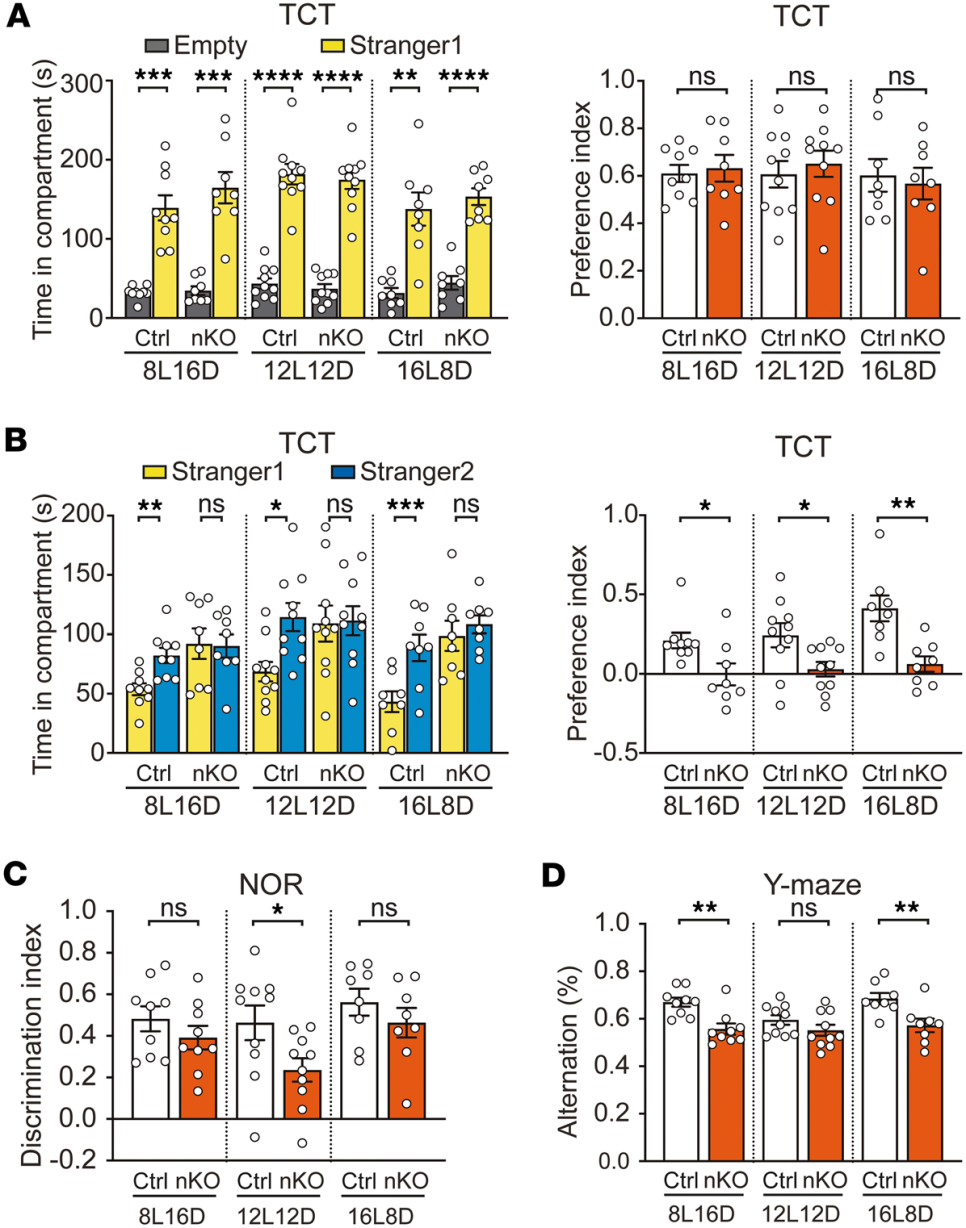
Autism-related behaviors in *Pogz*-deficient mice. (**A** and **B**) Three-chamber test for *Pogz^fl/fl^* (Ctrl) and *Pogz*-nKO mice under different photoperiod conditions. Social interaction (**A**, Left), social preference (**A**, Right), social novelty (**B**, Left), and novelty preference (**B**, Right) were calculated (*n* = 8–10). (**C**) Novel object recognition (NOR) test for *Pogz^fl/fl^* (Ctrl) and *Pogz*-nKO mice under different photoperiod conditions (*n* = 8–10). (**D**) Y-maze test for *Pogz^fl/fl^* (Ctrl) and *Pogz*-nKO mice under different photoperiod conditions (*n* = 8–10). All data in this figure are shown as mean ± SEM. Comparisons in **A** (left) and **B** (left) are conducted using paired 2-tailed Student’s *t* test, in **A** (right)**, B** (right)**, C**, and **D** using unpaired 2-tailed Student’s *t* test. ****P < 0.0001; ***P < 0.001; **P < 0.01; *P < 0.05.

Social behavior was evaluated using the 3-chamber test (TCT) under different photoperiod conditions. During the social interaction phase (second phase, sociability test), both *Pogz^fl/fl^* and *Pogz-*nKO mice spent more time interacting with the stranger mouse than the empty cage, with no significant difference in preference indices between genotypes (Figure 6A and Supplemental Figure 4, A–C), indicating normal sociability in nKO mice. In the third phase (social novelty preference test), both raw interaction times and the preference indices (Figure 6B and Supplemental Figure 4, A–C), showed that, unlike control mice, nKO mice failed to prefer the novel mouse over the familiar one. More importantly, in control mice, this preference was further enhanced under long photoperiod conditions (Supplemental Figure 4D). In contrast, *Pogz*-nKO mice failed to show a preference for the novel mouse under any photoperiod (Figure 6B and Supplemental Figure 4D). These findings suggest that POGZ is crucial for the recognition of novel social cues and plays an important role in the regulation of social novelty behavior in response to light.

Repetitive behaviors were assessed using grooming test, which showed no genotypic differences (Supplemental Figure 5). Cognitive performance was evaluated through the novel object recognition (NOR) and Y-maze test. Under 12l:12D conditions, *Pogz*-nKO mice exhibited a significantly lower discrimination index than controls in the NOR test (*P* < 0.05) (Figure 6C). However, this deficit was not observed under 8L16D or 16L8D conditions. In contrast, *Pogz*-nKO mice exhibited significantly reduced alternation percentages in the Y-maze test under 8L16D and 16L8D conditions, but not under12l:12D condition (Figure 6D).

Retinal function was evaluated using electroretinography (ERG). Across scotopic (0.001, 0.01, 0.1, 1.0, 3.0 cd·s/m^2^) conditions, both a-wave and b-wave amplitudes were comparable between WT and *Pogz*-nKO mice (Supplemental Figure 6, A–C), indicating normal retinal function. Wheel-running assays revealed that, although overall locomotor activity was reduced, circadian activity rhythms remained (Figure 3). In the 3-chamber test, *Pogz*-nKO mice showed comparable chamber exploration and baseline sociability across all photoperiods (Figure 6A). Together with previous reports indicating preserved nonsocial olfactory function in *Pogz*-deficient mice (46), these results demonstrate that retinal signalling, locomotor capacity, and general sensory function are sufficient for reliable measurement of circadian and social behaviors.

Overall, our behavior test suggests that photoperiod and POGZ may interact to influence ASD-related behaviors.

## Discussion

Our work has identified *POGZ*, an ASD-risk gene, as a circadian-related gene, with rhythmic expression in the SCN and liver, likely controlled by DBP via a conserved D-box motif in the *POGZ* proximal enhancer. We further demonstrate that POGZ interacts with and enhances CREB transcriptional activity, providing a mechanistic basis for the lengthened circadian period and impaired light entrainment observed in *Pogz*-deficient mice. These finding reveal a bidirectional interaction between POGZ and the circadian clock, suggesting that disruptions in this regulatory loop may contribute to sleep disturbances in patients with ASD who have POGZ mutations.

### D-box as an important regulatory element for POGZ gene.

The D-box is a critical yet relatively understudied circadian-relevant *cis-*regulatory element compared with the E-box and RORE motifs. It is activated by PAR bZIP family members, including Albumin D-site-Binding Protein (DBP), Thyrotroph Embryonic Factor (TEF), and Hepatic Leukemia Factor (HLF), while being repressed by the bZIP transcription factor E4BP4/NFIL3, forming an oscillatory transcriptional mechanism (53, 54). Studies in *Dbp*-KO mice show that D-box–driven gene expression remains rhythmic, likely due to compensatory activity from TEF and HLF (55). *Dbp/Tef/Hlf*–triple KO mice exhibit significant circadian period shortening (56), further highlighting the collective role of these factors in regulating circadian periodicity. Conversely, E4bp4 is the only known negative regulator of D-box, and its knockout disrupts circadian transcriptional outputs (53, 54, 57). A systematic study, by Yoshitane et al. delineated the genome-wide interplay between DBP and E4BP4 in the mouse liver. In total, 1,490 genomic regions showed overlapping binding by DBP and E4BP4. Genes targeted by both factors are more frequently arrhythmic in *E4bp4*-KO livers (20).

We identified a conserved D-box motif in the proximal enhancer of the POGZ gene and demonstrated that DBP promotes the *Pogz* expression through binding directly to this D-box element. However, no difference in *Pogz* expression pattern was observed in *Dbp*-KO mice (data not shown), likely due to compensation by TEF and HLF. Future studies using *Dbp*/*Tef*/*Hlf*–triple KO mice or *E4bp4*-KO mice, as well as AAV-mediated simultaneous manipulation of DBP, TEF, and HLF, would help to clarify the in vivo regulatory mechanisms controlling *Pogz*.

### POGZ as a regulator of circadian period.

The circadian period is a key parameter of the molecular clock, and mutations in core clock genes such as *Clock*, *Bmal1*, *Per1*, *Per2*, *Cry1*, and *Cry2* are known to disrupt circadian period length (58). For example, *Clock*^Δ19^ mutant mice exhibit an extended free-running period (~26–29 h) and eventually lose rhythmicity in constant darkness (DD) (59). Similarly, the *Cry1* and *Cry2* mutations exert opposing effects on period length, with *Cry1* KO shortening the period (~22.5 h), and *Cry2* KO lengthening it (~24.6 h) (60). Double KO of *Cry1* and *Cry2* abolishes rhythmicity, emphasizing their essential role in maintaining the integrity of the TTFL (60). Additionally, *Bmal1* KO leads to complete arrhythmicity, further demonstrating the central role of TTFL components in circadian regulation (58). The Period (*Per*) gene family also exhibits distinct roles: *Per1*- and *Per2*-KO mice exhibit shortened and lengthened periods, respectively, and their double KO results in complete loss of rhythmicity (61). In contrast, *Per3*-KO mice display minor reductions in period length and subtle alterations in circadian waveform (62). Casein Kinase 1 (CK1) is another crucial player in circadian regulation, with mutations in *Csnk1e* (*Tau* mutation) or *Csnk1d* significantly shortening the circadian period by accelerating PER protein degradation (63, 64). Additionally, the nuclear hormone receptors NR1Ds (NR1D1 and NR1D2, also known as REV-ERBα and -β) and RORs (RORα, RORβ, and RORγ), modulate circadian transcription through ROR elements (ROREs) present in core clock genes (65, 66). Mutations in *Nr1d1*, *Rora*, or *Rorb* also result in abnormal circadian period length (65, 67).

In this study, we show that *Pogz* deficiency subtly but significantly lengthens circadian period. This phenotype was consistently observed in 3 independent lines of *Pogz*-deficient mice under both DD and LL conditions. Importantly, small shifts in circadian periods can be physiologically meaningful, with temporal accumulation, as even subtle alterations may lead to phase misalignment between internal clocks and environmental cues. Such desynchronization has been associated with mood disorders, cognitive dysfunction, and metabolic disturbances (68–73). A potential mechanism underlying the regulation of POGZ on circadian period may involve its interaction with and modulation of CREB. Indeed, Wheaton et al. demonstrated that phosphorylation of CREB at Ser133 is crucial for circadian clock timing and light-induced entrainment in the SCN. In their knock-in (S/A CREB) mouse model, where Ser133 was mutated to alanine, they found that these mice exhibited a prolonged circadian period and impaired light-induced phase shifts, mirroring the phenotype observed in our *Pogz*-deficient mice (74).

### Involvement of POGZ in the light entrainment.

Light is the most powerful Zeitgeber for circadian entrainment, and its effects on phase shifts depending critically on the circadian time (CT) of exposure. Light exposure during the early subjective night (CT15–CT19) induces phase delays, while light during the late subjective night (CT22–CT2) results in phase advances (75, 76). The distinct responses are linked to differential transcriptional regulation of the *Per* genes: phase advances correspond to an acute induction of *Per1* transcription, resetting the clock forward, whereas phase delays involve stabilization of *Per2*, slowing down the clock without a significant increase in *Per1* expression (75).

At the molecular level, light stimuli activate the RHT, triggering the release of glutamate and pituitary adenylyl cyclase-activating polypeptide (PACAP) onto SCN neurons. These neurotransmitters initiate intracellular signaling cascades, including mitogen-activated protein kinase (MAPK) and cAMP pathways, leading to the phosphorylation of CREB. Phosphorylated CREB (pCREB) then binds to the CRE motif in the promoters of core circadian genes, such as *Per1* and *Per2*, regulating their expression in a phase-dependent manner (77).

In our study, we demonstrated diminished light-induced phase shift in *Pogz*-deficient mice, accompanied by reduced SCN c-Fos activation in response to light pulses, suggesting that POGZ plays a role in the light entrainment process. Moreover, our findings demonstrate that POGZ enhances CREB-mediated transcription, which may influence the sensitivity of the circadian clock to light. Although, the precise mechanisms through which POGZ enhances CREB transcriptional activity remain to be fully understood, it is conceivable that POGZ, as a chromatin remodeler (37), may modulate the accessibility of CREB and its cofactors to the promoters of genes such as *Per1* and *Per2*, thereby contributing to light-induced entrainment.

### Influence of photoperiod on behaviors.

Multiple studies have investigated the behavioral phenotypes of *Pogz*-deficient mice, yet their findings have been inconsistent, particularly regarding social interaction, anxiety-related behaviors, repetitive behaviors, and cognitive performance. Cunniff et al. reported no social deficits or cognitive impairments in *Pogz^+/–^* mice but observed reduced anxiety-like behaviors (78). Matsumura et al. found that an autism-associated *POGZ* mutation (*POGZ*^WT/Q1038R^) led to social deficits, reduced anxiety, and increased grooming behavior (37). Suliman-Lavie et al. found that *Pogz* Nestin^CRE^-cKO mice had spatial learning and working memory deficits, increased social approach behavior, but no changes in anxiety-related behaviors (46). These discrepancies may be attributed to differences in genetic models (i.e., KO, KI, cKO), behavioral paradigms, and environmental factors such as photoperiod and circadian timing.

Photoperiod, the length of daily light exposure, is a critical environmental cue that influences mood, cognition, and social behaviors. Variations in photoperiod are closely linked to neuropsychiatric conditions, particularly depression and anxiety-related behaviors (44). Seasonal changes in light exposure are well known to influence affective disorders, such as seasonal affective disorder (SAD). For instance, prolonged darkness is linked to increased depressive symptoms (77). In this study, we systematically examined *Pogz*-deficient mice under different photoperiod conditions.

We found that long photoperiod promoted social novelty preference in control mice; however, *Pogz*-nKO mice exhibited social deficits regardless of photoperiod conditions. Previous studies have consistently demonstrated that light exposure and photoperiod influence hippocampal and prefrontal cortical plasticity, as well as key neuromodulatory systems including BDNF, serotonin, dopamine, oxytocin, and vasopressin, all of which regulate social and cognitive behaviurs (79–83). These findings suggest that POGZ likely mediates the effects of photoperiod on social behavior by modulating these neural and neuromodulatory pathways. Together, our results highlight a critical role for POGZ in integrating environmental light cues to modulate neural circuits and social cognitive processes and provide a framework for future studies to dissect the underlying molecular and circuit-level mechanisms.

We also observed photoperiod- and paradigm-dependent alterations in memory performance in *Pogz*-nKO mice. Specifically, *Pogz*-nKO mice showed impaired memory under 12l:12D conditions in an NOR test, while exhibiting deficits under 16L8D and 8L16D conditions in a Y-maze test. These differences between paradigms may reflect the involvement of distinct neural circuits and cognitive processes. The NOR test primarily depends on perirhinal and prefrontal cortices, which support object recognition and novelty detection (84), whereas the Y-maze task relies more heavily on hippocampal-prefrontal networks underlying spatial working memory and exploratory behavior (85–87). Light exposure and circadian cues may differentially affect the excitability and plasticity of these regions, potentially contributing to the task-specific effects of *Pogz* deficiency. Although the precise mechanisms remain unclear, our findings underscore the convergent role of environmental cues (e.g., light cycles) and genetic vulnerabilities in regulating behaviors. These results highlight the importance of considering photoperiod as a crucial environmental factor in neurobehavioral studies, and future work should dissect the circuit- and molecular-level mechanisms by which POGZ integrates photic information to modulate cognitive performance.

In addition to circadian regulation, POGZ may also play a role in sleep-wake regulation. CREB, a key regulator of synaptic plasticity, plays a critical role in sleep homeostasis. As an example, Zhou et al. identified the LKB1–SIK3–HDAC4/5–CREB signaling pathway as a key regulator of sleep duration in mice (88). Given that POGZ enhances CREB transcriptional activity, it is plausible that POGZ influences both circadian and homeostatic processes in sleep via CREB-dependent mechanisms. It will be intriguing to explore the role and mechanism of POGZ in the homeostatic regulation of sleep.

## Methods

### Sex as a biological variable

In circadian behavioral testing, both male and female mice were included, and no sex differences were observed. For other behavioral tests, including cognitive and social behaviors, only male mice were used to minimize variability associated with the estrous cycle, ensuring more stable and interpretable results. We believe that the findings from these experiments are relevant to both sexes.

### Antibodies

The antibodies used in this study were as follows: anti-POGZ (ab167408, Abcam; A302-509A/A302-510A, Bethyl Laboratories), anti-CREB (9197, Cell Signaling Technology), anti-Phospho-CREB Ser133 (9198, Cell Signaling Technology), anti-HA (3724, Cell Signaling Technology), anti-Flag M2 (F3165, Sigma), anti-β-actin (66009-1-Ig, Proteintech) and anti-GAPDH (ab9485, Abcam), and c-Fos antibody (226003, 1:300, Synaptic Systems).

### Tissue sampling for rhythmic gene expression analysis

Tissues were collected at 6 time points (ZT0, ZT4, ZT8, ZT12, ZT16, ZT20) over a 24-h cycle to analyze the expression levels of *Pogz* and *Dbp*. Zeitgeber Time (ZT) 0 corresponds to lights-on, and ZT12 corresponds to lights-off in the 12:12-h light-dark cycle.

### Total RNA isolation and qPCR

Total RNA was extracted from tissues and cells using TRI Reagent Solution (AM9738, Invitrogen) following the manufacturer’s instructions. Complementary DNA (cDNA) synthesized using PrimeScript RT Reagent Kit (RR047, Takara) with 1 μg of RNA per reaction. qPCR analysis was performed on a LightCycler 96 Real-Time PCR System (Roche) with Hieff UNICON Universal Blue qPCR SYBR Green Master Mix (11184ES08, Yeasen). Gene expression levels were calculated using the 2^−ΔΔCt^ method, *Gapdh* gene was used as the internal control for normalization. Primers used for qPCR in this study are as follows: *Pogz* forward 5′-GCACAGAACAGCG-ACAGTAC-3′ and *Pogz* reverse 5′-CTGGACAGGTCTCAATACTGG-3′; *Dbp* forward 5′-GGAAACAGCAAGCCCAAAGAA-3′ and *Dbp* reverse 5′-CAGCGGCG-CAAAAAGACTC-3′; *Gapdh* forward 5′-AGGTCGGTGTGAACGGATTTG-3′ and *Gapdh* reverse 5′-TGTAGACCATGTAGTTGAGGTCA-3′.

### Protein extraction and western blot

Tissues and cells were lysed in RIPA buffer (150 mM NaCl, 1% Triton X-100, 0.1% SDS, 0.5% Sodium deoxycholate, 50 mM Tris-HCl), supplemented with protease and phosphatase inhibitors. Protein concentrations were determined using the BCA Protein Assay Kit (23227, Thermo Fisher). Equal amounts of protein (20–30 μg per lane) were separated by SDS-PAGE, transferred to PVDF membranes (IPVH00010, Millipore), blocked with 5% nonfat milk in TBST for 1 h, and incubated overnight with primary antibodies at 4°C. Membranes were incubated with HRP-conjugated secondary antibodies for 1 h at room temperature, developed with the ECL detection reagent (32106; ThermoScientific), and imaged using a ChemiDoc Imaging System (Bio-Rad). Densitometry was performed using ImageJ software, GAPDH or β-actin served as the loading control.

### Cell culture and transfection

Human osteosarcoma U-2OS cells (HTB-96, ATCC) were cultured in Dulbecco’s Modified Eagle’s Medium (DMEM; C11995500BT, Gibco) with 10% (vol/vol) fetal bovine serum (FBS; 10099-141C, Gibco) and 1% (vol/vol) penicillin/streptomycin (15140-122, Gibco) at 37°C and 5% CO_2_ in a humidified incubator. Cells were transfected with Lipo8000 Transfection Reagent (C0533FT, Beyotime) following the manufacturer’s instructions and were subjected to qPCR and Western blot analysis 48 h after transfection.

### ChIP assay

U2OS cells in 10 cm dish were transfected with the pcDNA3.1-DBP-HA plasmid (10 μg) using Lipo8000 Transfection Reagent (C0533FT, Beyotime) following the manufacturer’s instructions. After 48 h, cells were fixed with 1% formaldehyde at room temperature for 10 minutes and quenched with 0.125 M glycine for 5 minutes. Cells were sequentially lysed in Lysis Buffer 1 (50 mM Hepes-KOH, 140 mM NaCl, 1 mM EDTA, 10% glycerol, 0.5% NP-40, 0.25% Triton X-100) and Lysis Buffer 2 (10 mM Tris-HCl, 200 mM NaCl, 1 mM EDTA, 0.5 mM EGTA). Chromatin was then sonicatedto 200-500bp using a Misnoix 3000 sonicator in Lysis Buffer 3 (10 mM Tris-HCl, 100 mM NaCl, 1 mM EDTA, 0.5 mM EGTA, 0.1% sodium deoxycholate, 0.5% N-lauroylsarcosine).

For immunoprecipitation, sheared chromatin was incubated overnight at 4°C with anti-HA (3724, Cell Signaling Technology) and magnetic beads (88803, Thermo Fisher). Beads were washed with Wash Buffer (50 mM Hepes-KOH, 500 mM LiCl, 1 mM EDTA, 1% NP-40, 10% Na-Deoxycholate) to remove nonspecifically bound chromatin. Enriched DNA was eluted with Elution Buffer (50 mM Tris-HCl, 10 mM EDTA, 1% SDS) and subjected to crosslink reversal by RNase A treatment at 65°C for 4–5 h, followed by Proteinase K digestion at 55°C for 2 h. Purified DNA was analyzed by qPCR using primers targeting the D-box region of the *Pogz* promoter to confirm protein-DNA interactions. Primers used for ChIP-PCR in this study are as follows: Primer1 (P1) forward 5′-GTTTTGAGCGGGAGGAGGAA-3′ and P1 reverse 5′-CCGAGGGAGGAGACTTCGTA-3′; Primer2 (P2) forward: 5′-TTCCCGCTCCG-AGAAGGAGT-3′ and P2 reverse 5′-CCGAGGGAGGAGACTTCGT-3′; Primer3 (P3) forward 5′-TACGAAGTCTCCTCCCTCGG-3′ and P3 reverse 5′-GCGGCAGGAC-ATAATCACCA-3′; Primer4 (P4) forward 5′-GACCACAGGAGGTGAGTGAG-3′ and P4 reverse 5′-ATTGTCTCGCCTCTGAACGC-3′; Primer5 (P5) forward 5′-AGGCA-TTTACCCAACTAGAGGA-3′ and P5 reverse 5′-GCTCTCAGACCCTGA-GCAAT-3′.

### Dual luciferase reporter assay

Dual luciferase reporter assays were performed following the manufacturer’s protocol (E2920, Promega). In brief, cells in 24-well plates were transfected with 10 ng luciferase reporter and 1 ng Renilla per well. DBP (0, 20, 40 ng) or POGZ (0, 20, 40 ng) expression plasmids were added as indicated, and pcDNA3.1 was used to bring the total DNA amount to 200 ng per well. Luciferase and Renilla signals were measured using the SIRIUS Luminometer (Berthold Detection Systems).

### Animal models and housing conditions

*Pogz*–global KO mice were generated by Saiye Model Biology Research Center (Taicang) Co., LTD using CRISPRCas9 technology. Briefly, fertilized eggs from C57BL/6J mice were microinjected with sgRNAs and Cas9 mRNA and then implanted into pseudo-pregnant females. A founder (F0) mouse carrying a heterozygous 5891 bp deletion, resulting in the loss of *Pogz* exon 3 and 4, was identified (Supplemental Figure 3). This F0 mouse was bred with wild-type (WT) C57BL/6J mice to produce F1 heterozygotes, which were subsequently undercrossed to obtain F2 homozygous knockout mice. Genotyping was performed by PCR analysis of genomic DNA. The WT allele produced a 727 bp band, while the knockout allele generated a 431 bp band. The Primers used for *Pogz* global knockout genotyping are: 5′-GTTGGTGACCTAAC-TTTGTTGAGA-3′ (forward); 5′-GACAGTTTTCTGCTTCATTCTCCT-3′ (reverse).

The *Pogz^fl/fl^* mice were purchased from GemPharmatech Co. Ltd (Strain ID: T008148). Exons 4 and 9 of *Pogz* were flanked by *LoxP* sites to allow Cre recombinase-mediated excision. Conditional KO mice were generated by crossing *Pogz^fl/fl^* mice with *Nestin-Cre* (Stock No: 003771, Jackson Laboratory) or *UBC-Cre^ERT2^* (Stock No: 007179, Jackson Laboratory) mice to achieve tissue-specific or inducible deletion (tamoxifen treated), respectively. Before behavioral assays, mice were maintained under pathogen-free conditions with a 12:12-h light-dark cycle (lights on at 7:00 AM, lights off at 7:00 PM) at a constant temperature of 20°C ± 2°C, with ad libitum access to food and water. Genotyping was performed via PCR analysis of genomic DNA. The WT allele produced a 327 bp band, while the *loxP* allele showed a 431 bp band. The primers used for *Pogz* conditional knockout genotyping are: 5′-TCCTGACTGTGAAAACAGCATCTCC-3′ (forward); 5′-AAGCAGAAGGGACCA-AGTTTAGGG-3′ (reverse).

### Locomotor behavior

Mice aged 3–6 months were individually housed within cages equipped with running wheels, and ad libitum access to food and water. Locomotor activities were recorded as revolutions per 5-minute interval. Mice were initially entrained to a 12:12-h light-dark (LD) cycle (light intensity ~150 lux, lights on at 7:00 AM and lights off at 7:00 PM). After 2–3 weeks of activity recording under LD conditions, the mice were transferred to either constant darkness (DD) or constant light (LL) for approximately 4 weeks. To determine the light-induced phase shift of locomotor activity, mice were exposed to a 15-minute pulse of white light (150 lux) at CT 15 of constant darkness (DD). CT12 was designated as activity onset. Mice remained in their home cages but were transferred to a separate room for light exposure.

The light induced phase-shift amplitude was determined using regression lines drawn through the activity onset at least 7 days immediately before stimulation and 7 days after the reestablishment of a steady-state circadian period after stimulation. The free-run period and FFT were analyzed using ClockLab software (Actimetrics) in the Matlab environment. The free-run period was determined using a χ^2^ periodogram, analyzing data from days 10 to 25 under DD and LL conditions. Daily activity levels (total wheel revolutions) and FFT analysis were performed using data from the last 10 days under a LD cycle and days 10–25 under DD cycle. The FFT circadian amplitude was calculated as the peak relative amplitude within the circadian range (18–30 h), normalized to 100% total variance.

For the 8-h advance jet-lag experiments, the lights were turned off at 11:00 AM and turned back on at 11:00 PM on the first day. Mice were then maintained under this new light-dark schedule for 3 weeks. The onset of activity for each cycle was defined as the first concentrated bout of activity following an extended period of rest.

### Whole brain c-Fos labeling, imaging and analysis

The SOLID-DL 3D labeling protocol was applied in this study to label c-Fos^+^ cells throughout the whole brain (89). Briefly, fixed whole brains were incubated with the primary c-Fos antibody (226003, 1:300, Synaptic Systems) and secondary goat anti-rabbit IgG (H+L) secondary antibody, Alexa Fluor 594 (A-11037, 1:500, Thermo Fisher Scientific) for 7 days each. The brains were then counterstained with TO-PRO-3 (T3605, 1:1000, Thermo Fisher Scientific) for 5 days to label the cell nuclei. Cleared brains were imaged using light-sheet microscope (Lightsheet 7, Zeiss) equipped with a 5× objective lens (NA = 0.16). Image stacks were down-sampled using linear interpolation to reduce the data size. The Allen CCFv3 Brain Atlas with an isotropic voxel size of 25 μm was downloaded from the Allen website. The whole brain datasets were registered using the Elastix toolbox based on the cell nuclei channel. After registration, the SCN region was extracted from the whole mouse brain datasets. The following cell detecting and analysis were performed using Fiji software.

### Y2H

The pGBKT7-POGZ plasmid was constructed and transformed into the Y2H Gold yeast strain. It’s self-activation and toxicity were assessed, and POGZ expression was confirmed by Western blot. After verifying that the plasmid was nontoxic, had no self-activation and expressed POGZ correctly, the pGBKT7-POGZ yeast solution was mixed with a yeast AD library containing a human protein library. Hybrid yeast colonies were screened on SD/-Ade/-His/-Leu/-Trp/X-α-Gal/AbA plates, and blue colonies were selected. Plasmids from positive yeast clones were extracted, retransformed into *E*. *coli*, and ampicillin-resistant colonies were cultured for sequencing. The open reading frame (ORF) of the Gal4-AD sequence was analyzed to identify inserted sequences. Candidate proteins without frameshift mutations and with normal expression were selected as potential POGZ interactors.

### Co-IP

U2OS cells in 10 cm dishes were transfected with 5 μg POGZ-Flag and 5 μg CREB-HA plasmids (control groups received 5 μg pcDNA3.1 plasmids) and lysed in Co-IP buffer (50 mM Tris, 100 mM NaCl, 1 mM EDTA, 0.3% TritonX-100, 10% glycerol, pH 7.5), supplemented with a protease inhibitor cocktail. Protein complexes were immunoprecipitated overnight at 4°C using anti-Flag (F3165, Sigma) or anti-HA (3724, CST) antibodies. For endogenous Co-IP, cell lysates were incubated with anti-POGZ (A302-509A, Bethyl Laboratories) or anti-CREB (9197, CST) under the same conditions. Immunocomplexes were captured with Protein G Agarose Beads (P3296, Sigma), washed 3 times with lysis buffer, and analyzed by Western blot using anti-HA or anti-Flag for exogenous proteins, and anti-POGZ (A302-509A, Bethyl Laboratories) or anti-CREB (9197, CST) for endogenous proteins.

### Behavioral assessments

All behavioral tests, including social interaction and learning/memory assays, were performed at Zeitgeber time ZT9–ZT12 unless otherwise noted.

#### Three-chamber social interaction test (TCT).

The apparatus was an open-top plexiglass box measuring 62.8 cm × 42.5 cm × 22.2 cm, divided into 3 equally sized interconnected chambers (left, center, right). Mice were acclimated to the behavioral test room for at least 1 h prior to the first trial. During the habituation phase, 2 empty metal cages were placed in the left and right chambers, and the test mouse was introduced in the center chamber, allowed to explore freely for 10 minutes. In the subsequent social testing phase, an object was placed in one cage, while a C57BL/6 mouse (Stranger1, S1) of the same sex and age was placed in the other. The test mouse was returned to the center chamber for another 10 minutes exploration. In the social novelty phase, the object was replaced with a second C57BL/6 mouse (Stranger 2, S2), and the test mouse again was placed in the center chamber and allowed to explore for 10 minutes. Mouse behavior was recorded using automatic tracking software, and the novelty index was calculated as follows: Novelty index = (Time exploring S2 − Time exploring S1) / (Time exploring S2 + Time exploring S1).

#### Grooming.

A 6–8-week-old male mouse was placed in a transparent cylindrical container (20 cm in diameter and 30 cm in height) with a 1 cm-thick cushion layer at the bottom for comfort. The mouse acclimated to the container for 10 minutes, followed by a 10-minute observation period during which total grooming time was recorded. All observations and data collection were performed double-blind, and statistical analyses were conducted accordingly.

#### New Object Recognition (NOR).

The apparatus consisted of a square box measuring (25 × 25 × 40 cm; L ×W× H) and 3 objects approximately 5 cm in diameter. During the habituation phase, mice were placed in the center of the box and allowed to explore 2 identical objects, equally spaced from each other and the walls, for 10 minutes. Afterward, the mice were returned to their home cages for a 1-h rest. In the testing phase, 1 object was replaced with a novel object, and the mice were again placed in the center of the box to explore for 10 minutes. The time spent exploring the familiar (F) and the novel (N) objects was recorded, and the Recognition Index was calculated as follows: Recognition Index = (Time exploring novel object − Time exploring familiar object) / (Time exploring novel object + Time exploring familiar object).

#### Y-maze.

The apparatus was a Y-shaped maze comprising 3 identical arms (50 cm × 18 cm × 35 cm; L × W × H) radiating from a central area. Each mouse was placed at the center and allowed to freely explore all 3 arms for 10 minutes while its trajectory was recorded. A correct arm selection was defined as a continuous sequence in which the mouse entered each arms once without repetition; any repeated entry into the same arm was considered incorrect.

Distinct cohorts of mice were employed for behavioral assessments under each photoperiod condition.

### Electroretinogram (ERG)

ERG were performed as previously described (90). Briefly, mice were dark-adapted for 12 h under standardized conditions. Following anesthesia with sodium pentobarbital, pupils were dilated with compound tropicamide eye drops, and the corneas were kept moist with carbomer gel. ERG recordings were obtained using the RetiMINER system (AiErXi Medical Equipment Co. Ltd). A ground electrode was placed near the tail, a reference electrode was positioned subcutaneously, and a gold-loop electrode was placed on the cornea. All recordings were performed under dim red light. The a-wave amplitude was measured from baseline to the peak of the a-wave, and the b-wave amplitude was measured from the trough of the a-wave to the peak of the b-wave.

### Statistics

Statistical analysis was performed using SPSS 23.0 (SPSS, USA). One-way ANOVA, 2-way ANOVA, or a 2-tailed Student’s *t* test was used to assess significance. *P* < 0.05 was considered statistically significant. Data are presented as mean ± SEM with all data points representing biological replicates.

### Study approval

All in vivo procedures were approved by the IACUC of Central South University.

### Data availability

All data are available in the main text or the supplemental materials. All Supporting data values are available in the Supporting Data Values file.

## Author contributions

JDL and HL conceived and designed the research. TW, JH, CJX, PZ, YW, J Zhu, HL, J Zhang, and SZ performed the experiments and data analysis. JDL, HL, LZ, and CYL supervised the experiments. TW, HL, and JDL wrote the manuscript. All the authors read and approved the final manuscript. The two co-first authors contributed equally to this work. The order of their names was determined by mutual agreement among all authors based on their relative contributions to the study design, data analysis, and manuscript preparation.

## Conflict of interest

The authors have declared that no conflict of interest exists.

## Funding support

- National Natural Science Foundation of China, 32371218 (JDL).
- National Natural Science Foundation of China, 32400690 (HL).
- National Natural Science Foundation of China, 82401748 (YFW).
- Natural Science Foundation of Hunan Province, 2023SK2084 (JDL).
- Natural Science Foundation of Hunan Province, 2023RC4001 (JDL).
- Natural Science Foundation of Hunan Province, 2025JJ30041 (JDL).
- Natural Science Foundation of Hunan Province, 2025JJ60134 (HL.)
- Postdoctoral Fellowship Program of the China Postdoctoral Science Foundation, GZC20242039 (YFW).
- Postgraduate Scientific Research Innovation Project of Hunan Province, CX20210178 (TW).
- Postgraduate Scientific Research Innovation Project of Hunan Province, CX20230113 (JH).
- Independent Exploration and Innovation Project of Central South University, 1053320212326 (TW).
- Independent Exploration and Innovation Project of Central South University, 1053320241115 (PCZ).

## Supplementary Material

Supplemental data

Unedited blot and gel images

Supplemental video 1

Supplemental video 2

Supplemental video 3

Supplemental video 4

Supporting data values

## Version 1 03/17/2026

In-Press Preview

## Version 2 05/08/2026

Electronic publication

## References

[1] First MB (2013). Diagnostic and statistical manual of mental disorders, 5th edition, and clinical utility. J Nerv Ment Dis.

[2] Williams Buckley A (2020). Practice guideline: treatment for insomnia and disrupted sleep behavior in children and adolescents with autism spectrum disorder: Report of the Guideline Development, Dissemination, and Implementation Subcommittee of the American Academy of Neurology. Neurology.

[3] Veatch OJ (2017). Shorter sleep duration is associated with social impairment and comorbidities in ASD. Autism Res.

[4] Ballester P (2020). Sleep in autism: a biomolecular approach to aetiology and treatment. Sleep Med Rev.

[5] Missig G (2020). Sleep as a translationally-relevant endpoint in studies of autism spectrum disorder (ASD). Neuropsychopharmacology.

[6] Ballester P (2019). Sleep problems in adults with autism spectrum disorder and intellectual disability. Autism Res.

[7] Bruni O (2025). Sleep and circadian disturbances in children with neurodevelopmental disorders. Nat Rev Neurol.

[8] Yang DF (2023). Acute sleep deprivation exacerbates systemic inflammation and psychiatry disorders through gut microbiota dysbiosis and disruption of circadian rhythms. Microbiol Res.

[9] Martinez-Cayuelas E (2024). Sleep problems and circadian rhythm functioning in autistic children, autism with co-occurring attention deficit hyperactivity disorder, and typically developing children: A comparative study. Autism.

[10] Goldstein AN (2014). The role of sleep in emotional brain function. Annu Rev Clin Psychol.

[11] Walker MP (2009). Overnight therapy? The role of sleep in emotional brain processing. Psychol Bull.

[12] Rasch B (2013). About sleep’s role in memory. Physiol Rev.

[13] Mazurek MO (2019). Course and predictors of sleep and co-occurring problems in children with autism spectrum disorder. J Autism Dev Disord.

[14] Richdale AL (2009). Sleep problems in autism spectrum disorders: prevalence, nature, & possible biopsychosocial aetiologies. Sleep Med Rev.

[15] Logan RW (2019). Rhythms of life: circadian disruption and brain disorders across the lifespan. Nat Rev Neurosci.

[16] Hirano A (2016). The intricate dance of post-translational modifications in the rhythm of life. Nat Struct Mol Biol.

[17] Takahashi JS (2017). Transcriptional architecture of the mammalian circadian clock. Nat Rev Genet.

[18] Ko CH (2006). Molecular components of the mammalian circadian clock. Hum Mol Genet.

[19] Partch CL (2014). Molecular architecture of the mammalian circadian clock. Trends Cell Biol.

[20] Yoshitane H (2019). Functional D-box sequences reset the circadian clock and drive mRNA rhythms. Commun Biol.

[21] Kohtala S (2021). Time is of the essence: coupling sleep-wake and circadian neurobiology to the antidepressant effects of ketamine. Pharmacol Ther.

[22] Hastings MH (2018). Generation of circadian rhythms in the suprachiasmatic nucleus. Nat Rev Neurosci.

[23] Quintero JE (2003). The biological clock nucleus: a multiphasic oscillator network regulated by light. J Neurosci.

[24] Sakamoto K (2013). Clock and light regulation of the CREB coactivator CRTC1 in the suprachiasmatic circadian clock. J Neurosci.

[25] Antle MC (2005). Orchestrating time: arrangements of the brain circadian clock. Trends Neurosci.

[26] Sun X (2023). POGZ suppresses 2C transcriptional program and retrotransposable elements. Cell Rep.

[27] Sun X (2022). Autism-associated protein POGZ controls ESCs and ESC neural induction by association with esBAF. Mol Autism.

[28] Satterstrom FK (2020). Large-scale exome sequencing study implicates both developmental and functional changes in the neurobiology of autism. Cell.

[29] Nagy D (2022). Genotype-phenotype comparison in POGZ-related neurodevelopmental disorders by using clinical scoring. Genes (Basel).

[30] White J (2016). POGZ truncating alleles cause syndromic intellectual disability. Genome Med.

[31] Stessman HAF (2016). Disruption of POGZ is associated with intellectual disability and autism spectrum disorders. Am J Hum Genet.

[32] Pascolini G (2020). A novel patient with White-Sutton syndrome refines the mutational and clinical repertoire of the POGZ-related phenotype and suggests further observations. Am J Med Genet A.

[33] Merriweather A (2022). A novel, de novo intronic variant in POGZ causes White-Sutton syndrome. Am J Med Genet A.

[34] Assia Batzir N (2020). Phenotypic expansion of POGZ-related intellectual disability syndrome (White-Sutton syndrome). Am J Med Genet A.

[35] Mudassir BU (2025). Denovo variants in POGZ and YY1 genes: The novel mega players for neurodevelopmental syndromes in two unrelated consanguineous families. PLoS One.

[36] Marquezini BP (2024). Exploring the molecular pathways linking sleep phenotypes and *POGZ*-associated neurodevelopmental disorder. J Med Genet.

[37] Matsumura K (2020). Pathogenic POGZ mutation causes impaired cortical development and reversible autism-like phenotypes. Nat Commun.

[38] Moore RY (1972). Loss of a circadian adrenal corticosterone rhythm following suprachiasmatic lesions in the rat. Brain Res.

[39] Moore RY (2002). Suprachiasmatic nucleus organization. Cell Tissue Res.

[40] Wen S (2020). Spatiotemporal single-cell analysis of gene expression in the mouse suprachiasmatic nucleus. Nat Neurosci.

[41] Gudmundsdottir B (2018). POGZ is required for silencing mouse embryonic β-like hemoglobin and human fetal hemoglobin expression. Cell Rep.

[42] Sharma VK (1999). Relationship between light intensity and phase resetting in a mammalian circadian system. J Exp Zool.

[43] Lee B (2010). CREB influences timing and entrainment of the SCN circadian clock. J Biol Rhythms.

[44] Fernandez DC (2018). Light affects mood and learning through distinct retina-brain pathways. Cell.

[45] Markenscoff-Papadimitriou E (2021). Autism risk gene POGZ promotes chromatin accessibility and expression of clustered synaptic genes. Cell Rep.

[46] Suliman-Lavie R (2020). Pogz deficiency leads to transcription dysregulation and impaired cerebellar activity underlying autism-like behavior in mice. Nat Commun.

[47] Parvataneni T (2020). Perspective on melatonin use for sleep problems in autism and attention-deficit hyperactivity disorder: a systematic review of randomized clinical trials. Cureus.

[48] Dai X (2025). The role of circadian rhythms and sleep in the aetiology of autism spectrum disorder and attention-deficit/hyperactivity disorder: new evidence from bidirectional two-sample Mendelian randomization analysis. Autism.

[49] Moyses-Oliveira M (2023). Genetic basis of sleep phenotypes and rare neurodevelopmental syndromes reveal shared molecular pathways. J Neurosci Res.

[50] Yang Z (2016). Circadian-relevant genes are highly polymorphic in autism spectrum disorder patients. Brain Dev.

[51] Nicholas B (2007). Association of Per1 and Npas2 with autistic disorder: support for the clock genes/social timing hypothesis. Mol Psychiatry.

[52] Liu D (2023). Autistic-like behavior and cerebellar dysfunction in Bmal1 mutant mice ameliorated by mTORC1 inhibition. Mol Psychiatry.

[53] Mitsui S (2001). Antagonistic role of E4BP4 and PAR proteins in the circadian oscillatory mechanism. Genes Dev.

[54] Ueda HR (2005). System-level identification of transcriptional circuits underlying mammalian circadian clocks. Nat Genet.

[55] Lopez-Molina L (1997). The DBP gene is expressed according to a circadian rhythm in the suprachiasmatic nucleus and influences circadian behavior. EMBO J.

[56] Gachon F (2004). The loss of circadian PAR bZip transcription factors results in epilepsy. Genes Dev.

[57] Chen M (2022). E4BP4 coordinates circadian control of cognition in delirium. Adv Sci (Weinh).

[58] Bunger MK (2000). Mop3 is an essential component of the master circadian pacemaker in mammals. Cell.

[59] Vitaterna MH (1994). Mutagenesis and mapping of a mouse gene, Clock, essential for circadian behavior. Science.

[60] van der Horst GT (1999). Mammalian Cry1 and Cry2 are essential for maintenance of circadian rhythms. Nature.

[61] Bae K (2001). Differential functions of mPer1, mPer2, and mPer3 in the SCN circadian clock. Neuron.

[62] Shearman LP (2000). Targeted disruption of the mPer3 gene: subtle effects on circadian clock function. Mol Cell Biol.

[63] Lowrey PL (2000). Positional syntenic cloning and functional characterization of the mammalian circadian mutation tau. Science.

[64] Meng QJ (2008). Setting clock speed in mammals: the CK1 epsilon tau mutation in mice accelerates circadian pacemakers by selectively destabilizing PERIOD proteins. Neuron.

[65] Preitner N (2002). The orphan nuclear receptor REV-ERBalpha controls circadian transcription within the positive limb of the mammalian circadian oscillator. Cell.

[66] Sato TK (2004). A functional genomics strategy reveals Rora as a component of the mammalian circadian clock. Neuron.

[67] Zhang EE (2009). A genome-wide RNAi screen for modifiers of the circadian clock in human cells. Cell.

[68] Emens JS (2024). Mood correlates with circadian alignment in healthy individuals. Sleep Health.

[69] Walker WH (2020). Circadian rhythm disruption and mental health. Transl Psychiatry.

[70] Scheer FA (2009). Adverse metabolic and cardiovascular consequences of circadian misalignment. Proc Natl Acad Sci U S A.

[71] Tamura N (2024). Longitudinal course and outcome of social jetlag in adolescents: a 1-year follow-up study of the adolescent sleep health epidemiological cohorts. J Sleep Res.

[72] Levandovski R (2011). Depression scores associate with chronotype and social jetlag in a rural population. Chronobiol Int.

[73] Roenneberg T (2012). Social jetlag and obesity. Curr Biol.

[74] Wheaton KL (2018). The phosphorylation of CREB at serine 133 is a key event for circadian clock timing and entrainment in the suprachiasmatic nucleus. J Biol Rhythms.

[75] Schwartz WJ (2011). Distinct patterns of Period gene expression in the suprachiasmatic nucleus underlie circadian clock photoentrainment by advances or delays. Proc Natl Acad Sci U S A.

[76] Ashton A (2022). Photic entrainment of the circadian system. Int J Mol Sci.

[77] Brenna A (2021). PER2 mediates CREB-dependent light induction of the clock gene Per1. Sci Rep.

[78] Cunniff MM (2020). Altered hippocampal-prefrontal communication during anxiety-related avoidance in mice deficient for the autism-associated gene *Pogz*. Elife.

[79] Soler JE (2018). Light modulates hippocampal function and spatial learning in a diurnal rodent species: a study using male nile grass rat (Arvicanthis niloticus). Hippocampus.

[80] Miller MA (2015). Photoperiod is associated with hippocampal volume in a large community sample. Hippocampus.

[81] Deats SP (2015). Hypothalamic dopaminergic neurons in an animal model of seasonal affective disorder. Neurosci Lett.

[82] Santoso P (2018). Suprachiasmatic vasopressin to paraventricular oxytocin neurocircuit in the hypothalamus relays light reception to inhibit feeding behavior. Am J Physiol Endocrinol Metab.

[83] Lazzerini Ospri L (2024). Light affects the prefrontal cortex via intrinsically photosensitive retinal ganglion cells. Sci Adv.

[84] (2020). Object recognition memory: distinct yet complementary roles of the mouse CA1 and perirhinal cortex. Front Mol Neurosci.

[85] Bai W (2024). Hippocampal-prefrontal high-gamma flow during performance of a spatial working memory. Brain Res Bull.

[86] Ruggiero RN (2024). Dysfunctional hippocampal-prefrontal network underlies a multidimensional neuropsychiatric phenotype following early-life seizure. Elife.

[87] Young RA (2025). Hippocampal-prefrontal communication subspaces align with behavioral and network patterns in a spatial memory task. eNeuro.

[88] Zhou R (2022). A signalling pathway for transcriptional regulation of sleep amount in mice. Nature.

[89] Zhu J (2024). SOLID: minimizing tissue distortion for brain-wide profiling of diverse architectures. Nat Commun.

[90] Zou J (2023). Melatonin protects against NMDA-induced retinal ganglion cell injury by regulating the microglia-TNFα-RGC p38 MAPK pathway. Int Immunopharmacol.

