# Supplementary material for: Reciprocal regulation between autism risk gene POGZ and circadian clock
Source: JCI Insight. 2026 Mar 17;11(9):e193622. doi: 10.1172/jci.insight.193622 (PMC13232009; doi:10.1172/jci.insight.193622)
Supplement: Supplemental data [file jciinsight-11-193622-s034.pdf]

Supplementary data for

Reciprocal regulation between autism risk gene *POGZ* and circadian clock

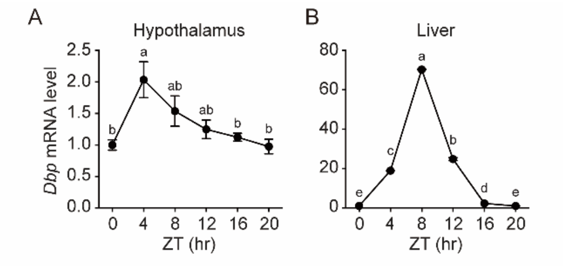

Supplemental Figure 1. Rhythmic oscillation of *Dbp* expression in multiple tissues. (A-B) qRT-PCR analysis reveals the rhythmic oscillation of *Dbp* mRNA levels in the hypothalamus (A) and liver (B) across a 24-hour light-dark (LD) cycle. Data are presented as mean ± s.e.m. (n = 3). Letters (a, b, ab in **A**, a, b, c, d, e in **B**) indicate statistical differences between time points ($P < 0.05$), determined by one-way ANOVA followed by Tukey's post-hoc test and annotated using the standard CLD method: points sharing at least one letter are not significantly different, whereas points with no shared letters differ significantly.

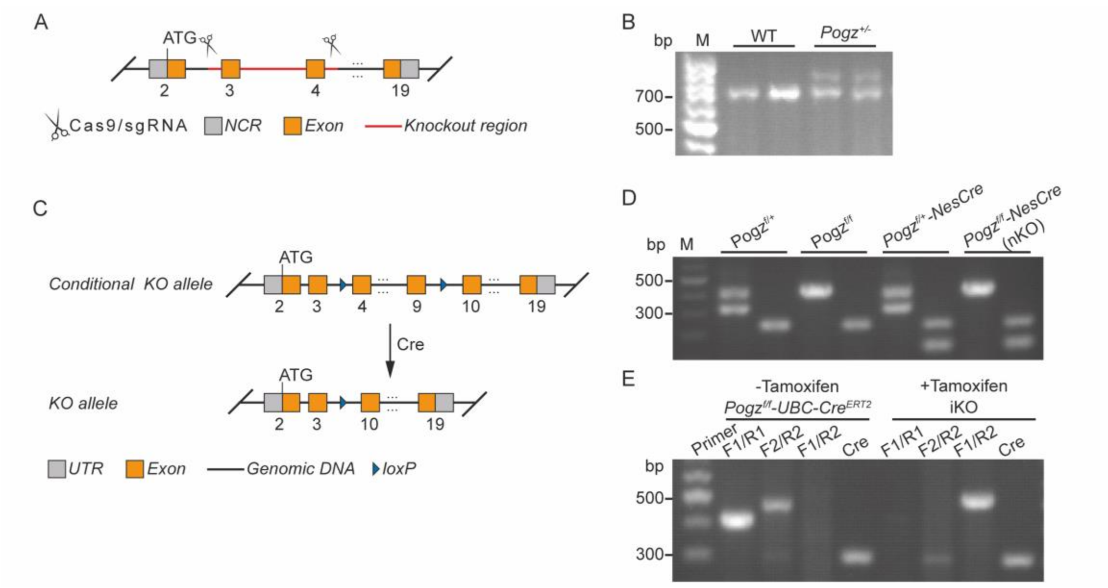

Supplemental Figure 2. Validation of *Pogz* conventional and conditional knockout mouse models. (A) Schematic of the strategies for generating the *Pogz* global knockout (KO) mice. (B) Genotyping results confirming the successful generation of *Pogz* heterozogous KO mice. (C) Schematic of the approach for generating *Pogz* conditional knockout mice. (D-E) Genotyping results confirming the successful generation of *Pogz* nkO and *Pogz* iKO (Tamoxifen treated) mice.

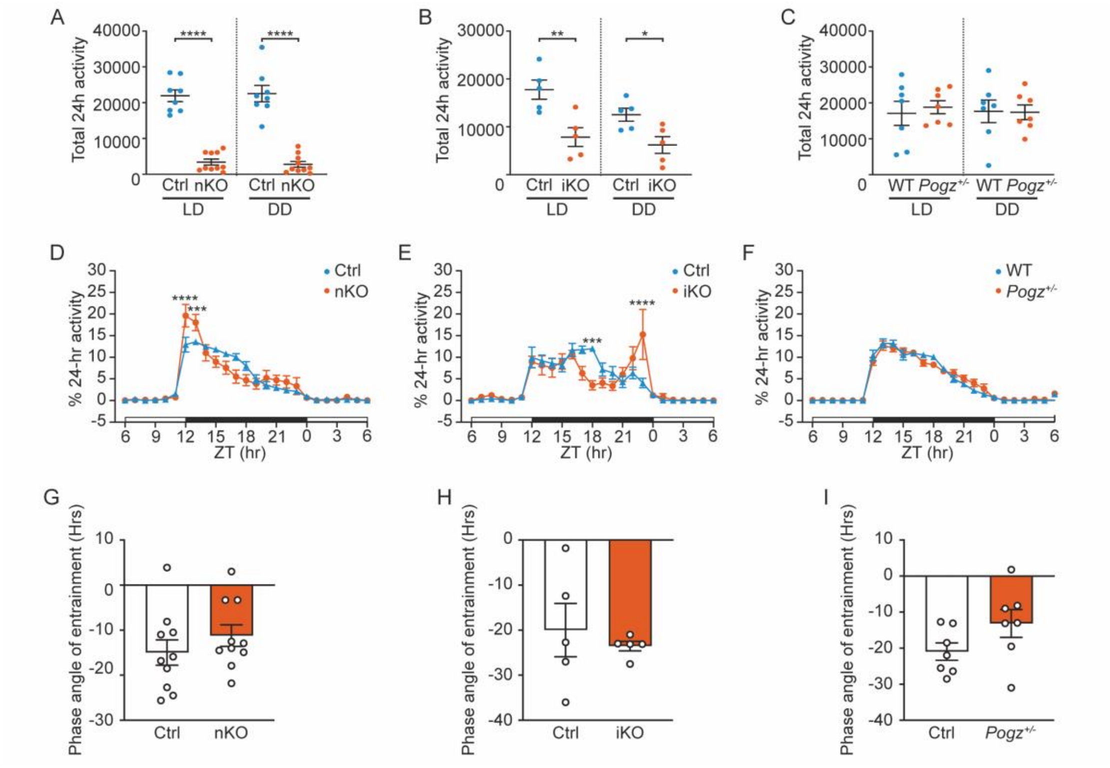

Supplemental Figure 3. Locomotor activity rhythms in *Pogz*-deficient mice under LD and DD conditions. (A-C) Total 24-hour locomotor activity in *Pogz* nKO (A), *Pogz* iKO (B), and *Pogz* global knockout (KO) (C) mice, compared to controls under LD and DD conditions. Data are presented as mean ± s.e.m. (n = 5-10). (D-F) Percentage of 24-hour activity across Zeitgeber times (ZT) in *Pogz* nKO (D), *Pogz* iKO (E), and *Pogz* KO (F) mice, compared to controls. (G-I) Phase angle of entrainment to the lighting cycle in *Pogz* nKO (G), *Pogz* iKO (H), and $Pogz^{+/-}$ (I) mice. Phase angle of entrainment was calculated as the time difference between lights-off and nocturnal activity onset, averaged over five consecutive days. Statistical analysis: unpaired, two-tailed Student's t-test was used for panels A–C and G–I; two-way ANOVA was used for panels D–F. Significance levels are indicated as follows: ****p < 0.0001, ***p < 0.001.

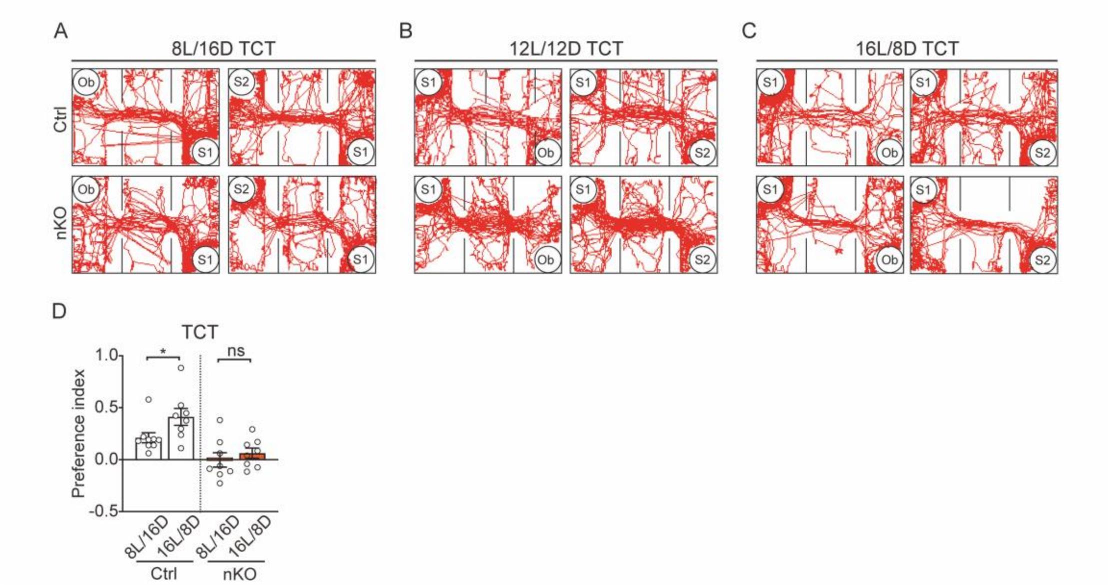

Supplemental Figure 4. Social behaviors of *Pogz*-deficient mice under different photoperiods. (A-C) Three-chamber test of $Pogz^{f/f}$ (Ctrl) and *Pogz* nKO mice under 8L/16D (A), 12L/12D (B), and 16L/8D (C) conditions. Objects (Ob), social targets (S1), or novel social targets (S2). (D) Comparison of the social novelty preference index in Ctrl and nKO mice under 8L/16D and 16L/8D photoperiods. Data are presented as mean ± s.e.m. (n = 8-10). Statistical analysis: unpaired two-tailed Student's t-test, $^{*}p < 0.05$.

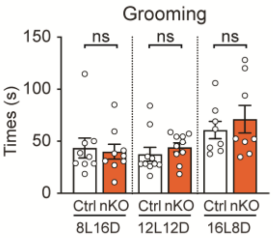

Supplemental Figure 5. Stereotypic behaviors of *Pogz*-deficient mice under different photoperiods**.** Grooming test of *Pogz*$^{f/f}$ (Ctrl) and *Pogz* nKO mice under 8L/16D, 12L12D and 8L/16D conditions. Data are presented as mean ± s.e.m. (n = 8-10). Statistical analysis: unpaired two-tailed Student's t-test, ns: p >0.05.

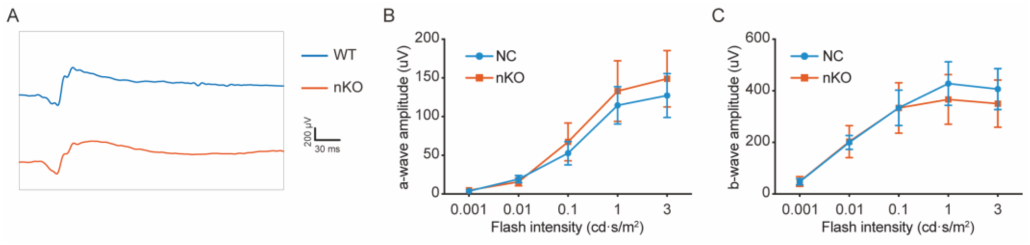

Supplemental Figure 6. No significant difference in retinal function was observed between wild-type and *Pogz* nKO mice. (A) Representative ERG waveforms from one eye of a single mouse in the wild-type and nKO groups. (B-C) Amplitudes of the scotopic ERG a-wave (B) and b-wave (C) in both groups across different flash intensities. Data are presented as mean ± s.e.m. (n = 4-6). Statistical analysis: two-way ANOVA.
