# Supplementary material for: Reciprocal regulation between autism risk gene POGZ and circadian clock
Source: JCI Insight. 2026 Mar 17;11(9):e193622. doi: 10.1172/jci.insight.193622 (PMC13232009; doi:10.1172/jci.insight.193622)

Figure 1D

Anti-POGZ

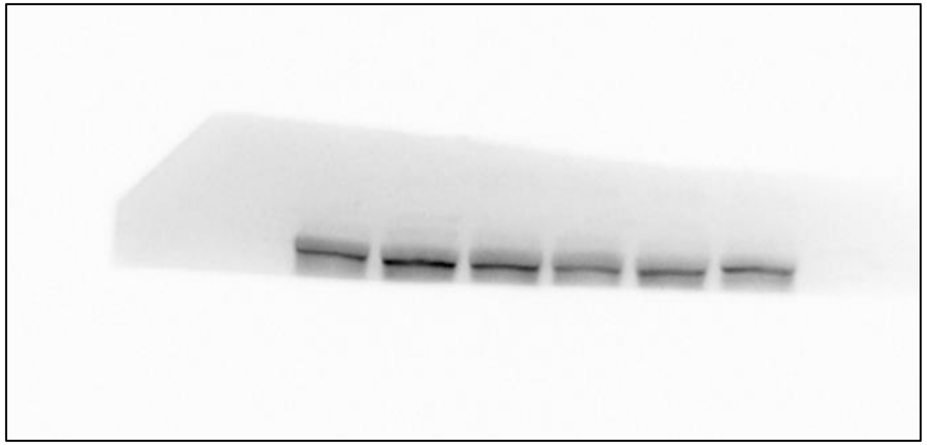

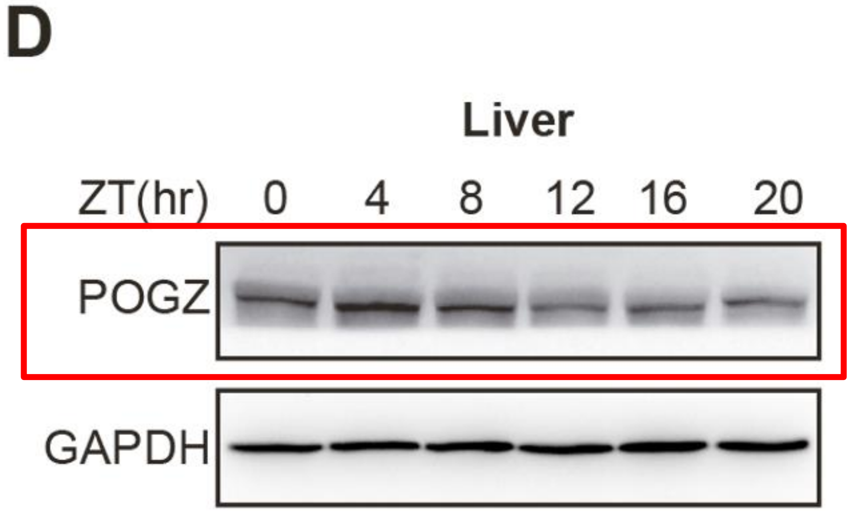

Figure 1D

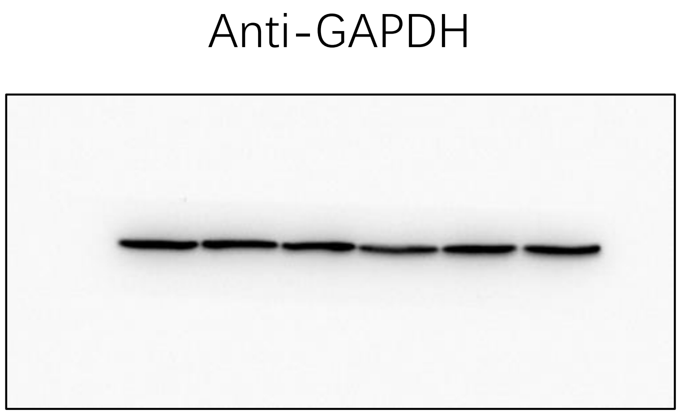

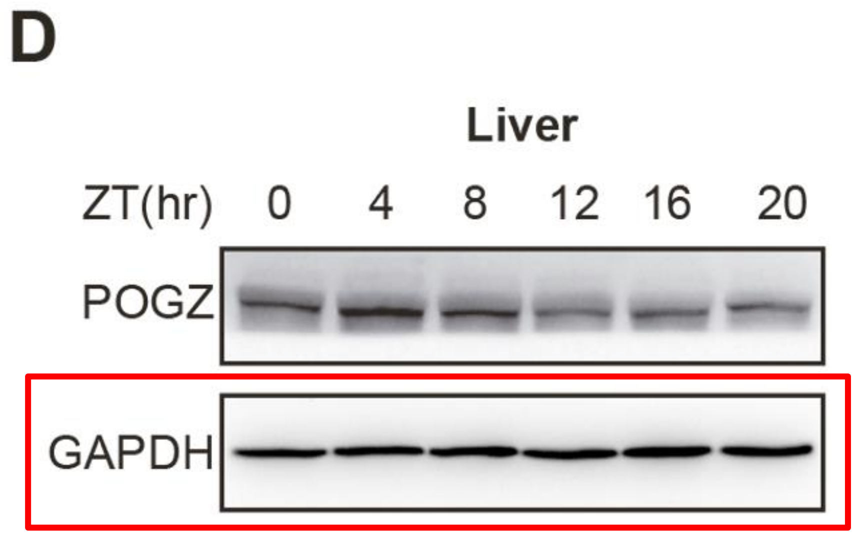

Figure 2B

Anti-POGZ

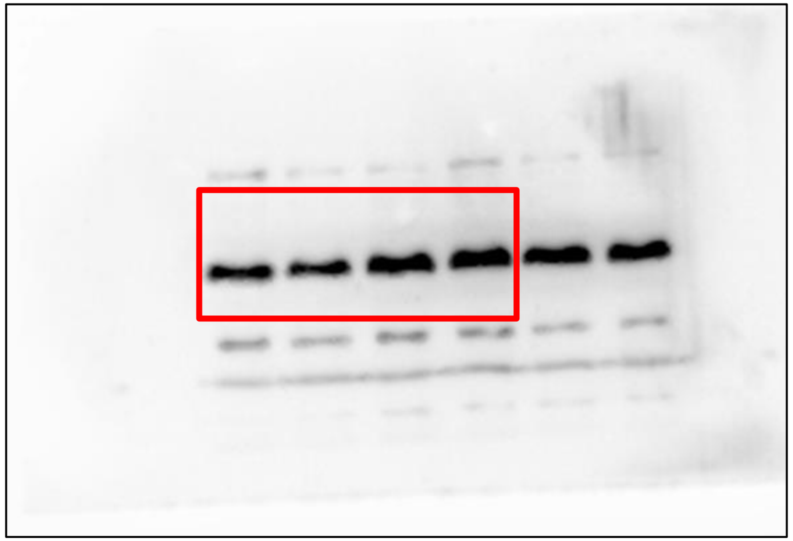

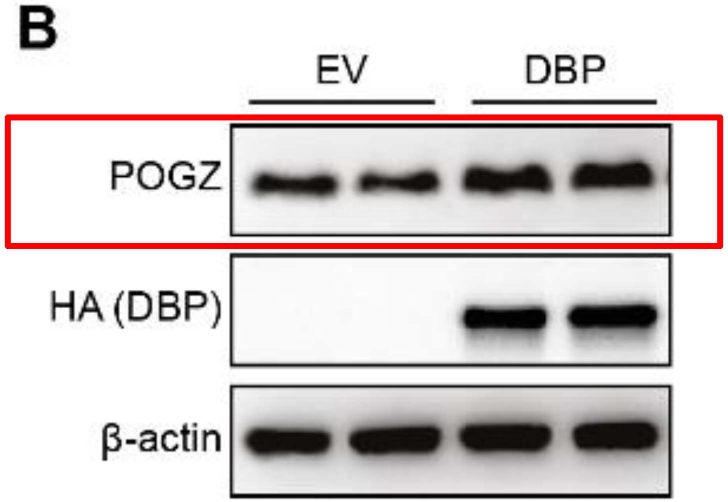

Figure 2B

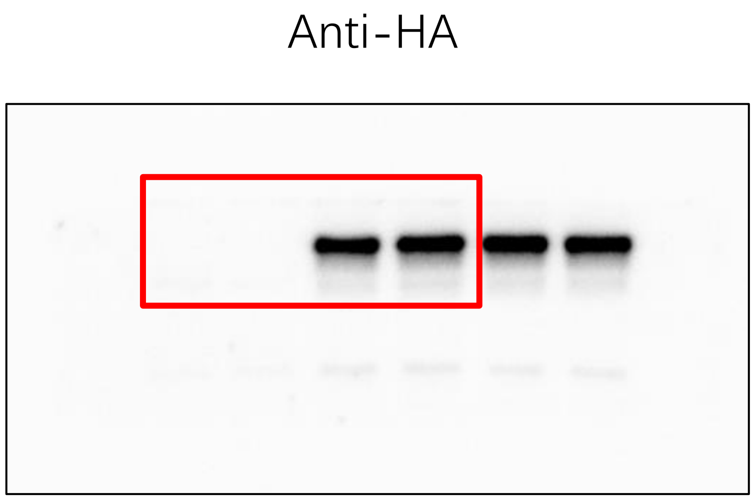

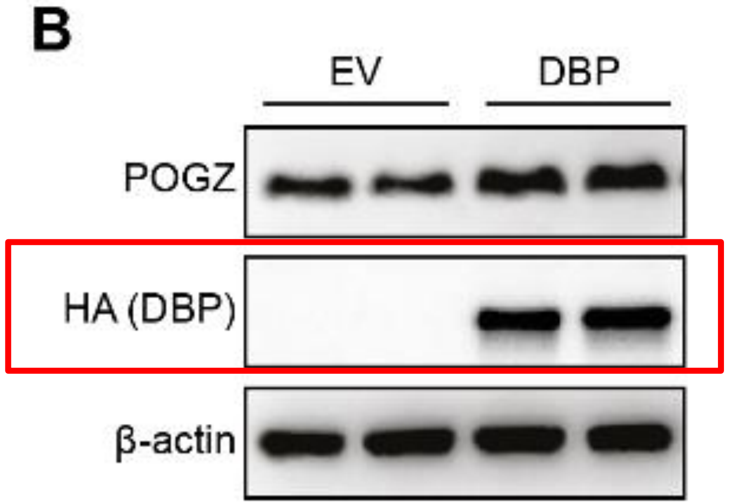

Figure 2B

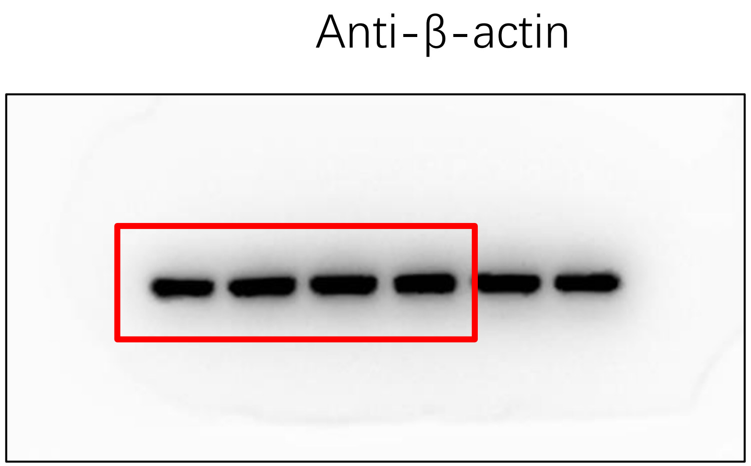

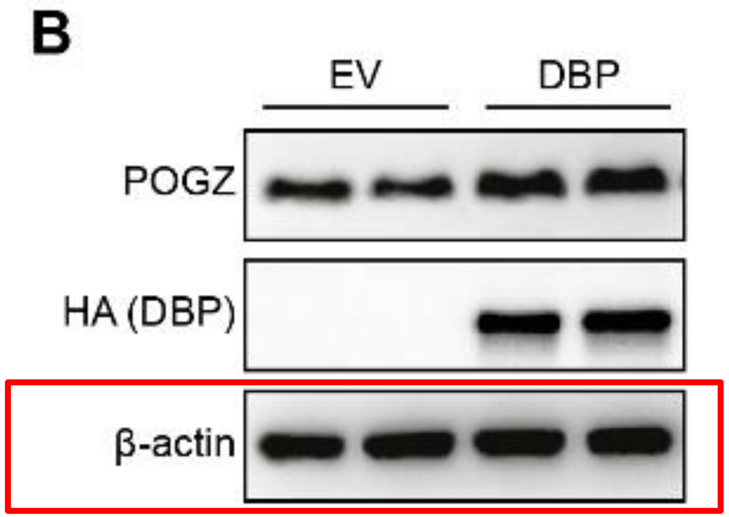

Figure 2E

Anti-POGZ

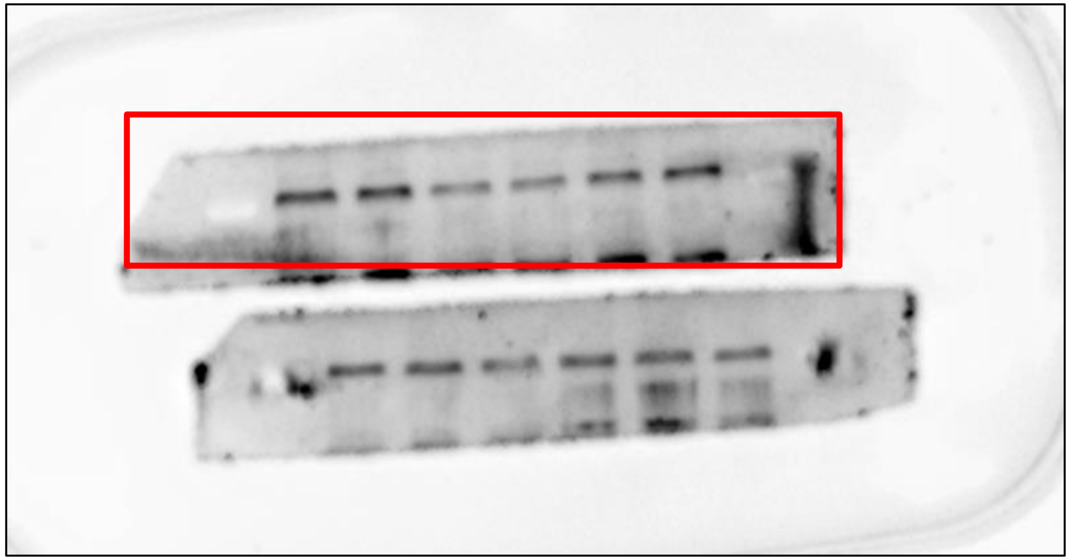

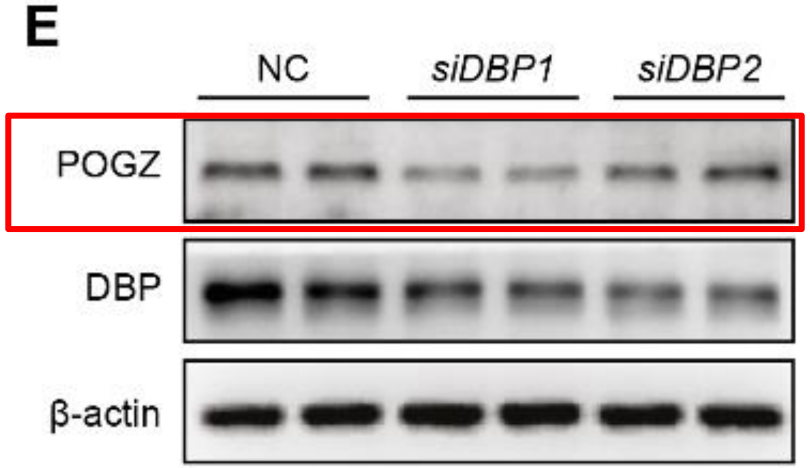

Figure 2E

Anti-DBP

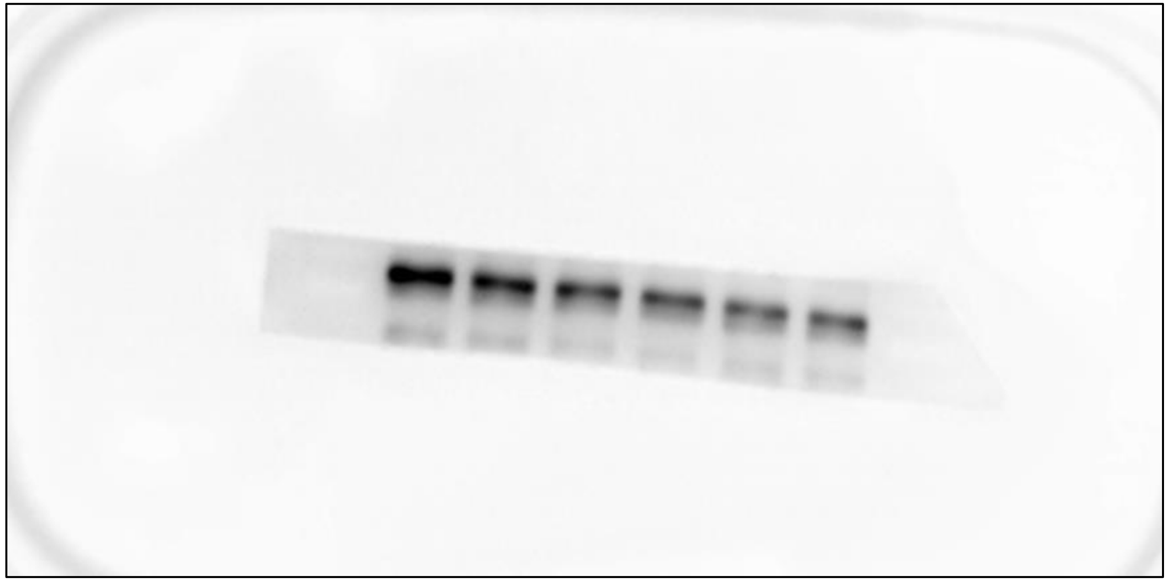

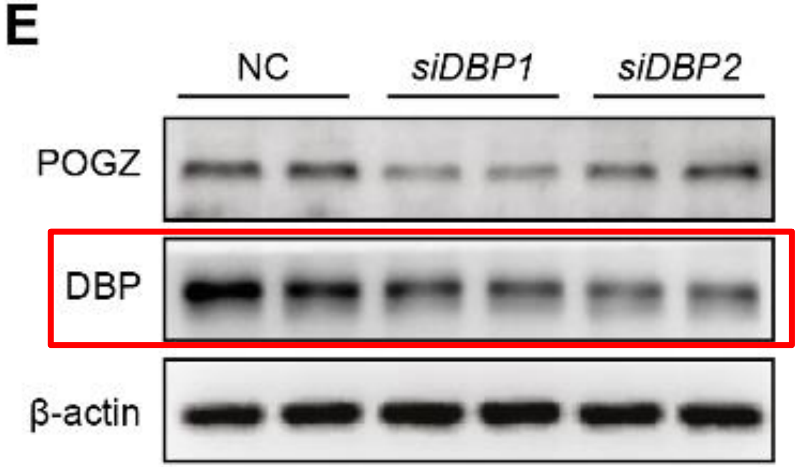

Figure 2E

Anti-β-actin

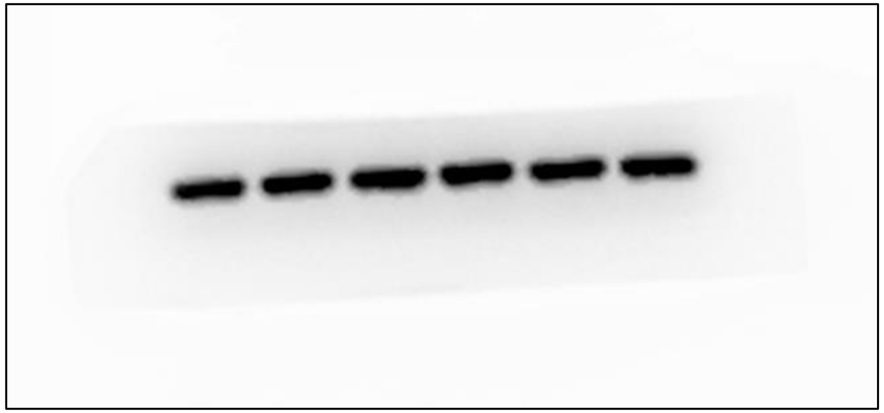

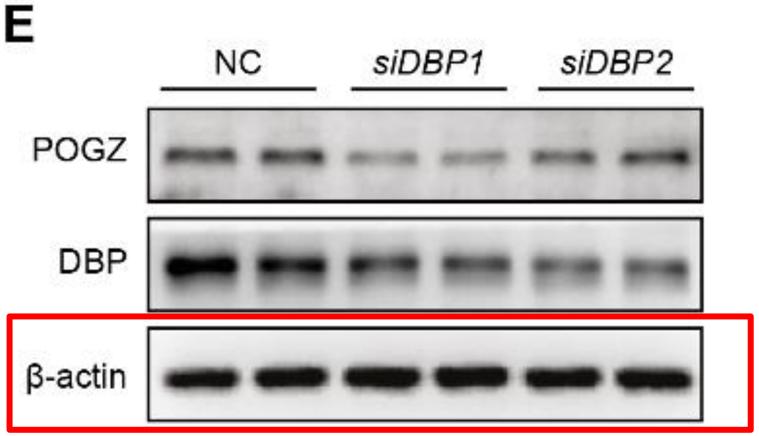

Figure 5B

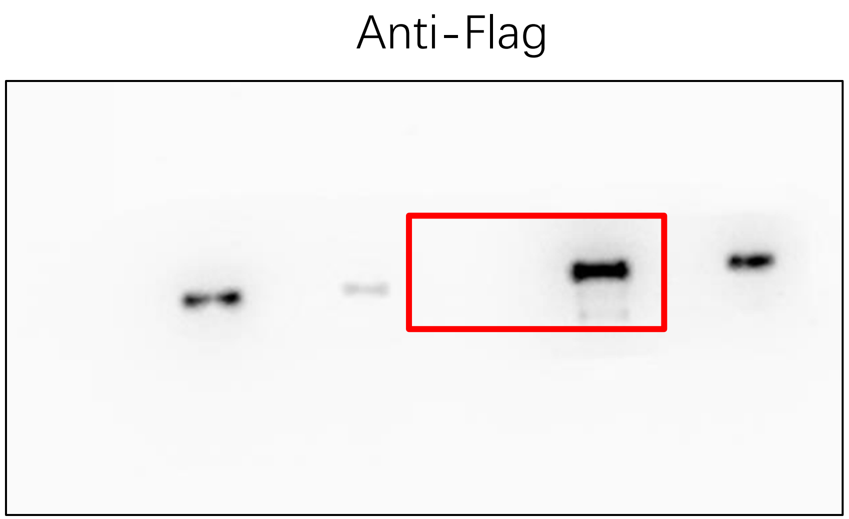
Anti-Flag

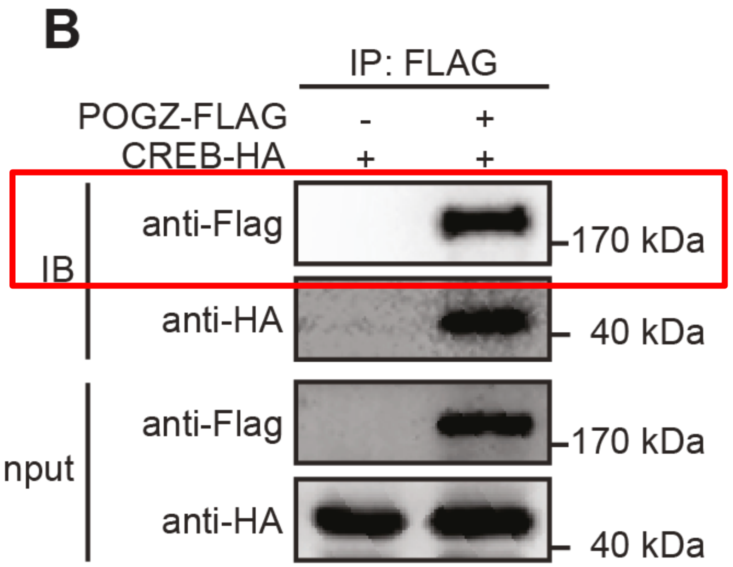
B
IP: FLAG
POGZ-FLAG - +
CREB-HA + +
IB
anti-Flag
170 kDa
anti-HA
40 kDa
nput
anti-Flag
170 kDa
anti-HA
40 kDa

Figure 5B

Anti-HA

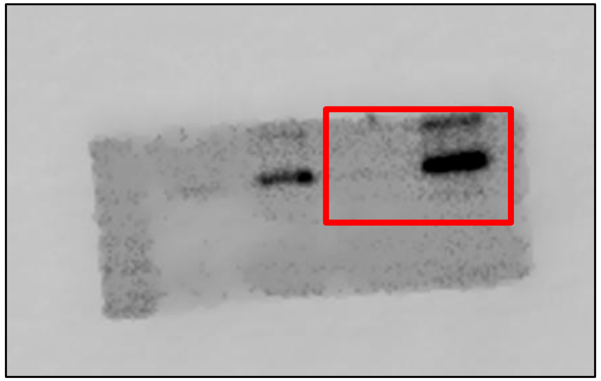

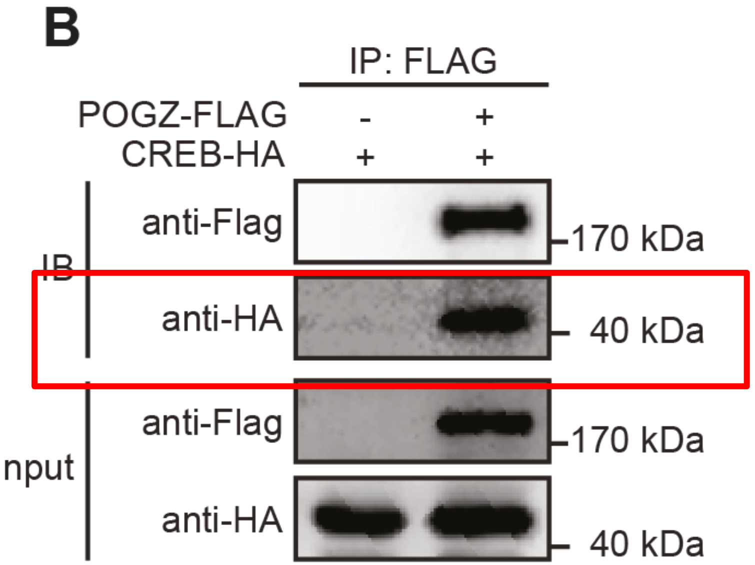

Figure 5B

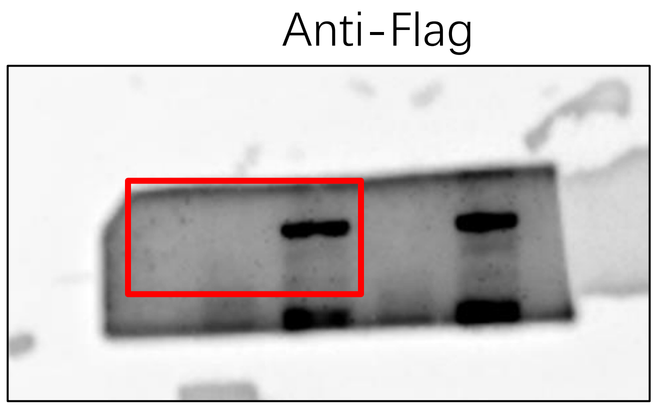

Anti-Flag

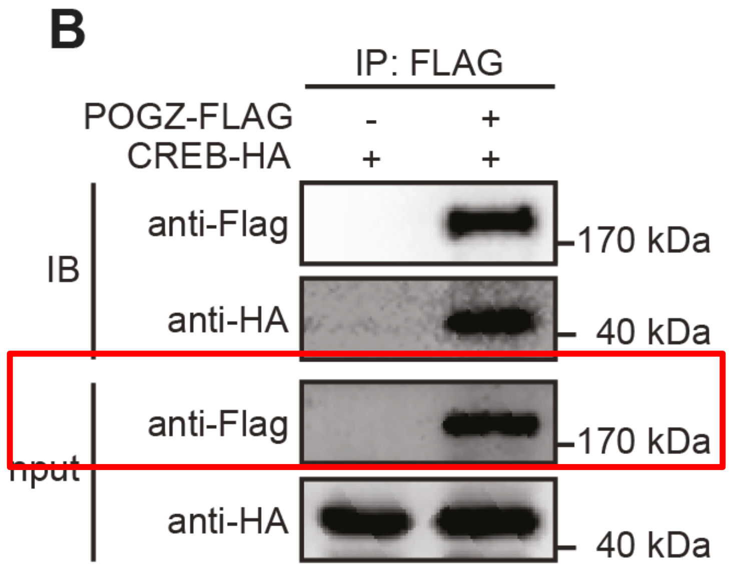

B
IP: FLAG
POGZ-FLAG - +
CREB-HA + +
IB
anti-Flag
170 kDa
anti-HA
40 kDa
anti-Flag
170 kDa
anti-HA
40 kDa

Figure 5B

Anti-HA

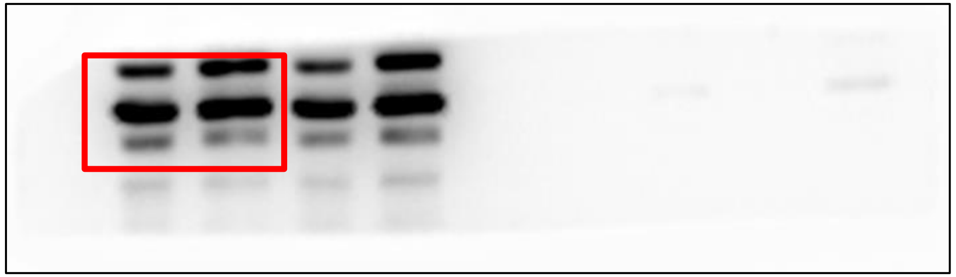

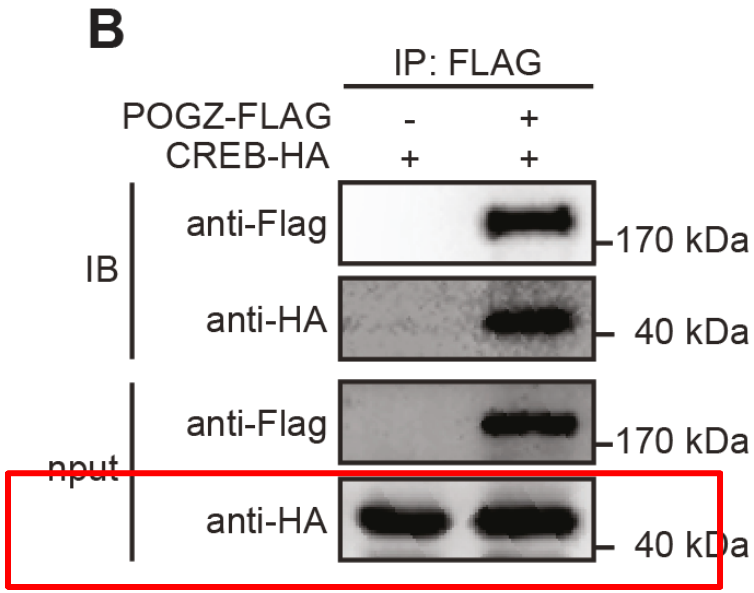

Figure 5C

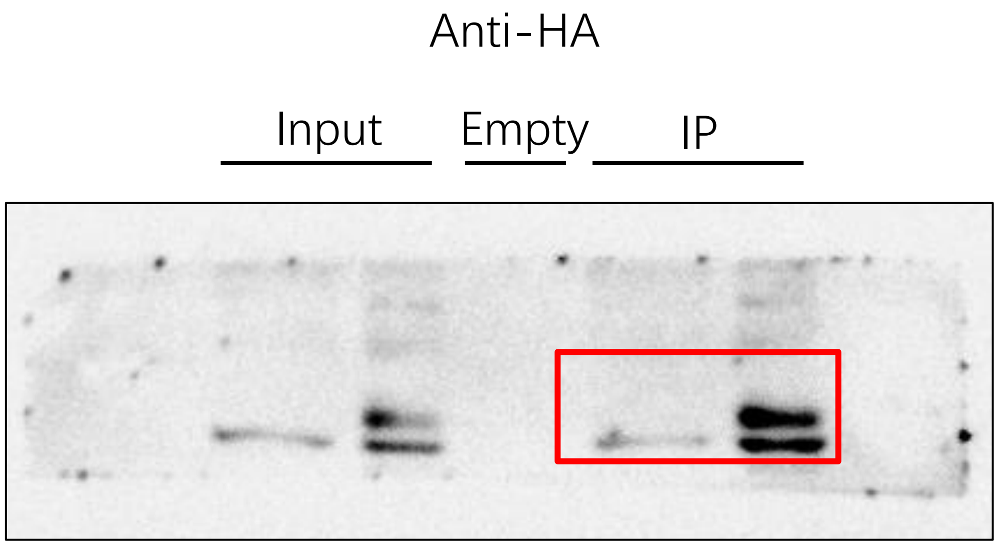
Anti-HA
Input
Empty
IP

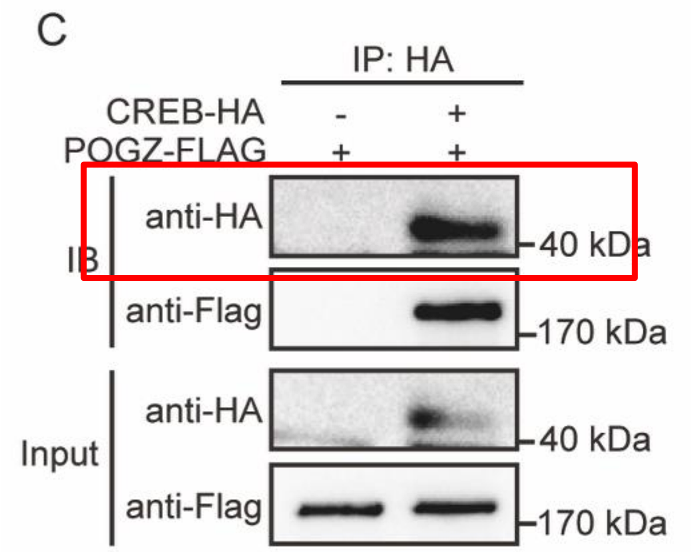
C
IP: HA
CREB-HA - +
POGZ-FLAG + +
IB
anti-HA
40 kDa
anti-Flag
170 kDa
Input
anti-HA
40 kDa
anti-Flag
170 kDa

Figure 5C

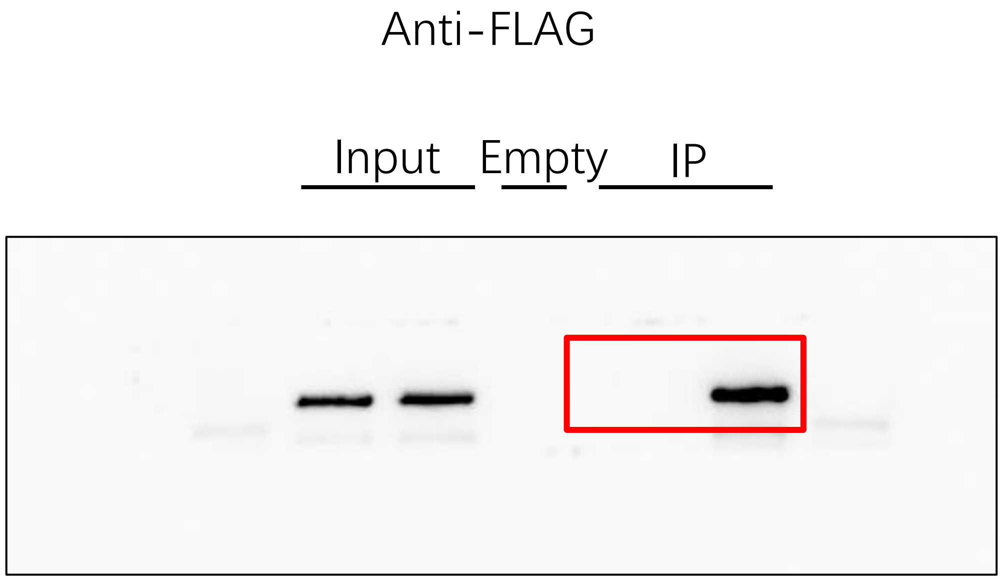

Anti-FLAG
Input
Empty
IP

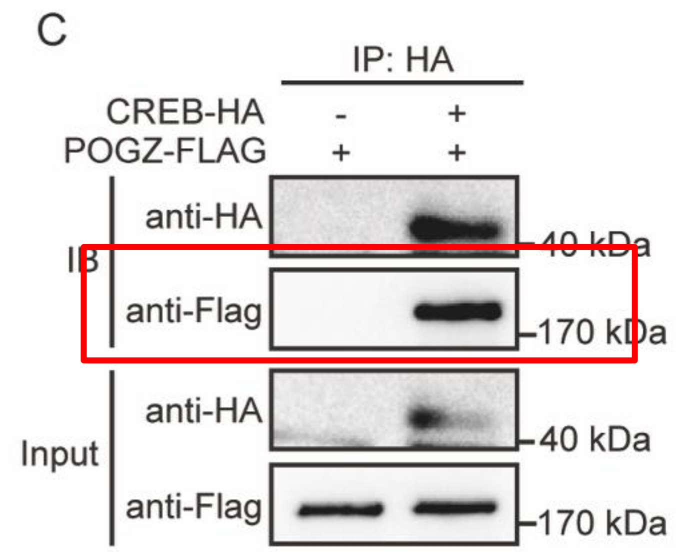

C
IP: HA
CREB-HA - +
POGZ-FLAG + +
IB
anti-HA
40 kDa
anti-Flag
170 kDa
Input
anti-HA
40 kDa
anti-Flag
170 kDa

Figure 5C

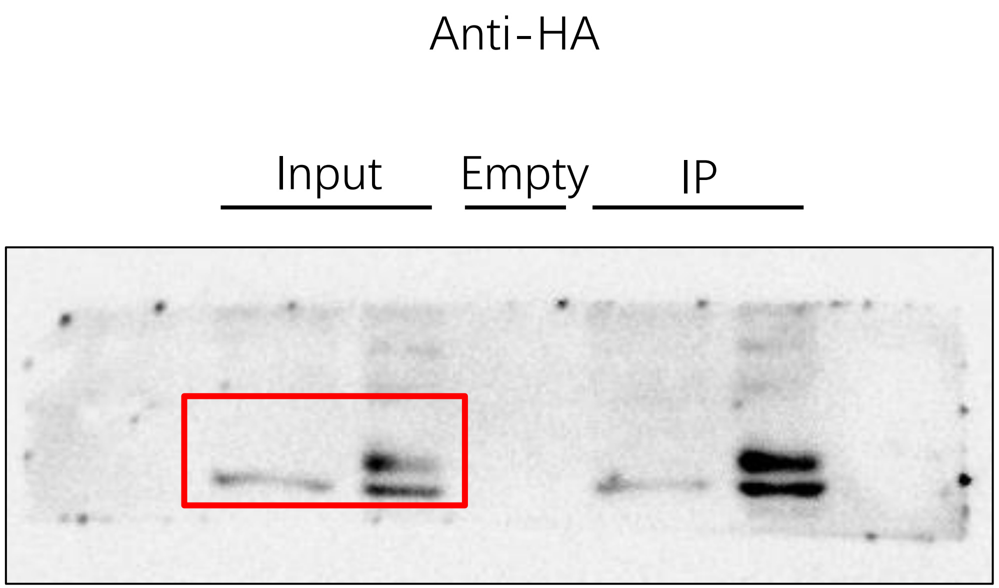

Anti-HA
Input
Empty
IP

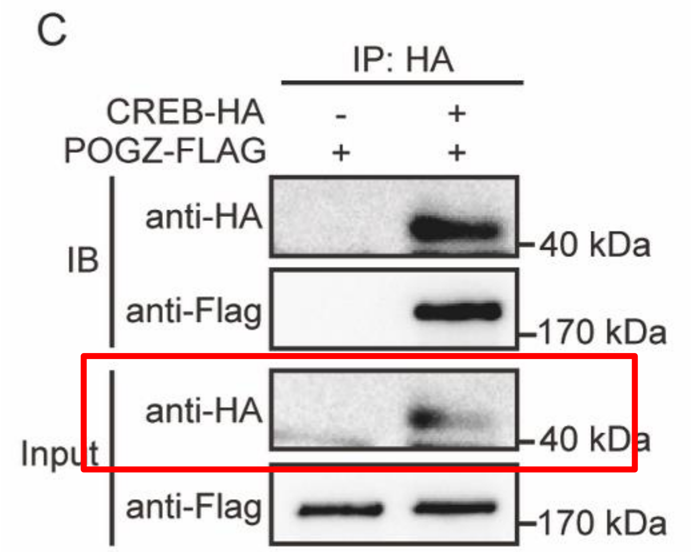

C
IP: HA
CREB-HA
POGZ-FLAG
-
+
+
+
IB
anti-HA
anti-Flag
Input
anti-HA
anti-Flag
40 kDa
170 kDa
40 kDa
170 kDa

Figure 5C

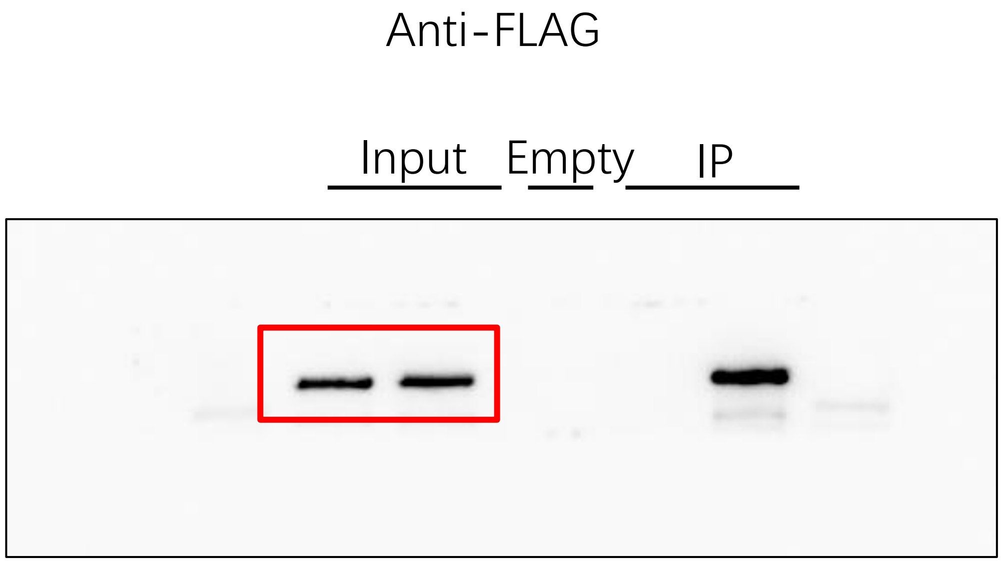

Anti-FLAG
Input
Empty
IP

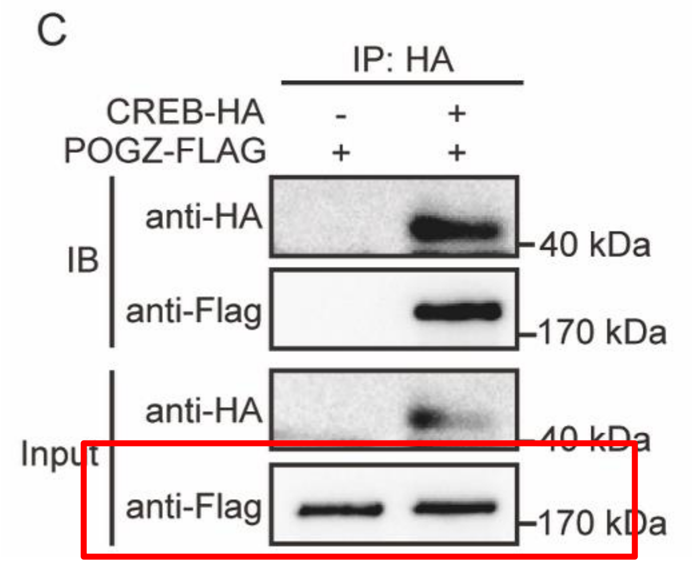

C
IP: HA
CREB-HA
POGZ-FLAG
IB
anti-HA
anti-Flag
Input
anti-HA
anti-Flag
40 kDa
170 kDa
40 kDa
170 kDa

Figure 5D

Anti-POGZ

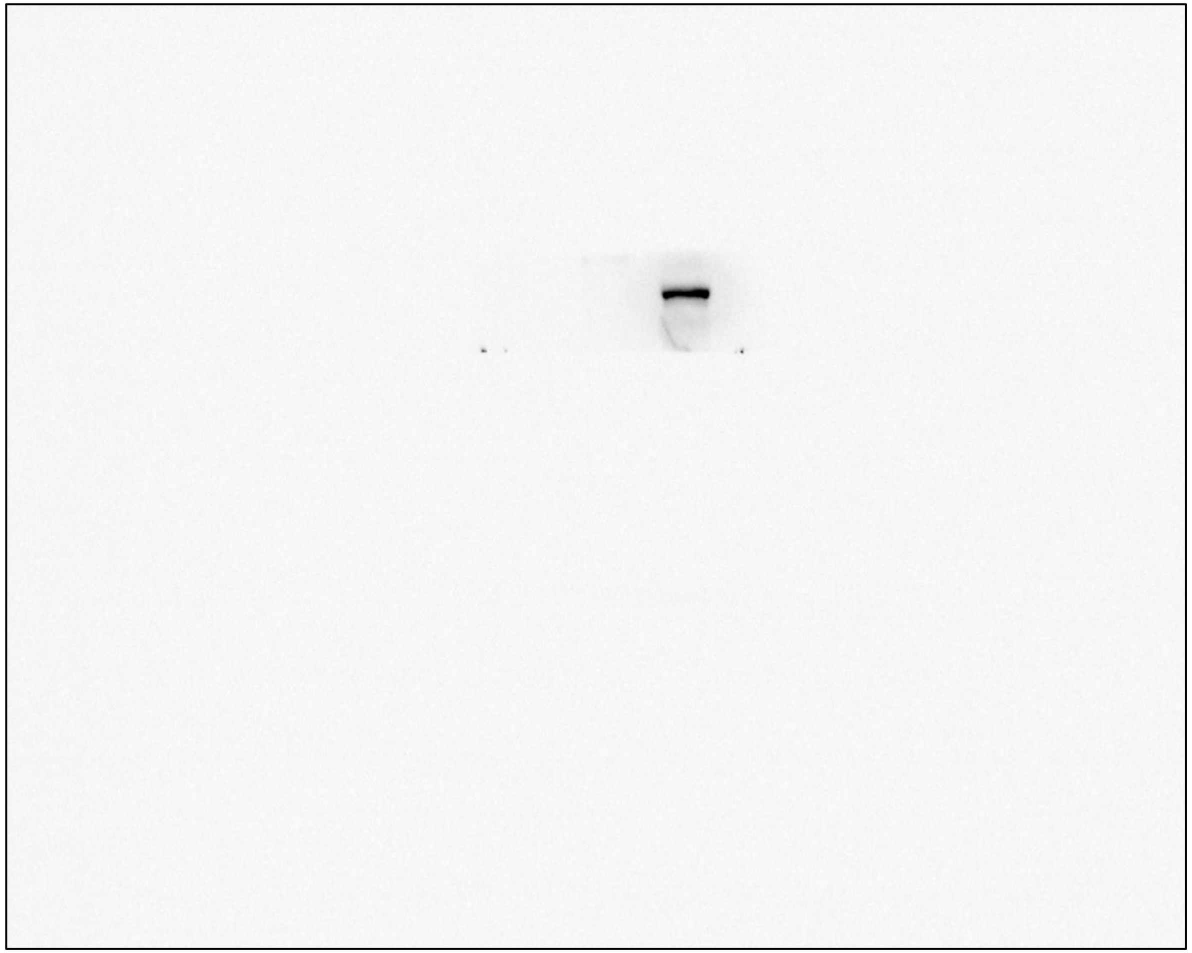

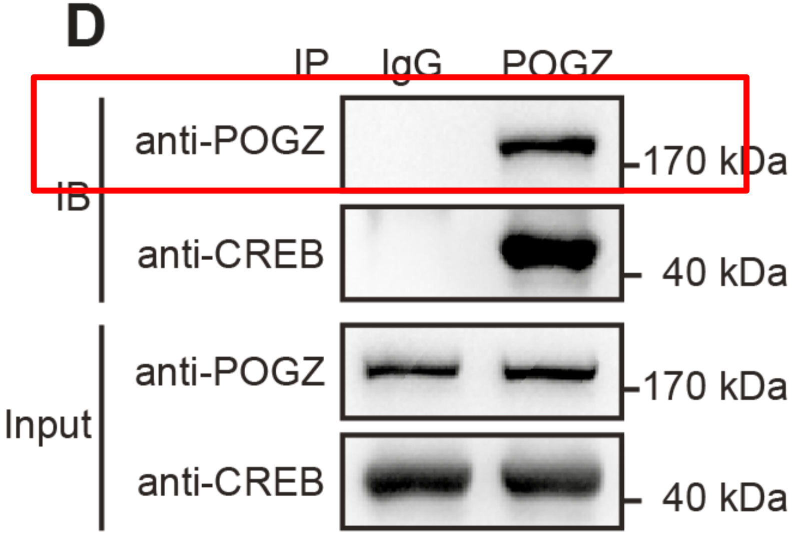

Figure 5D

Anti-CREB

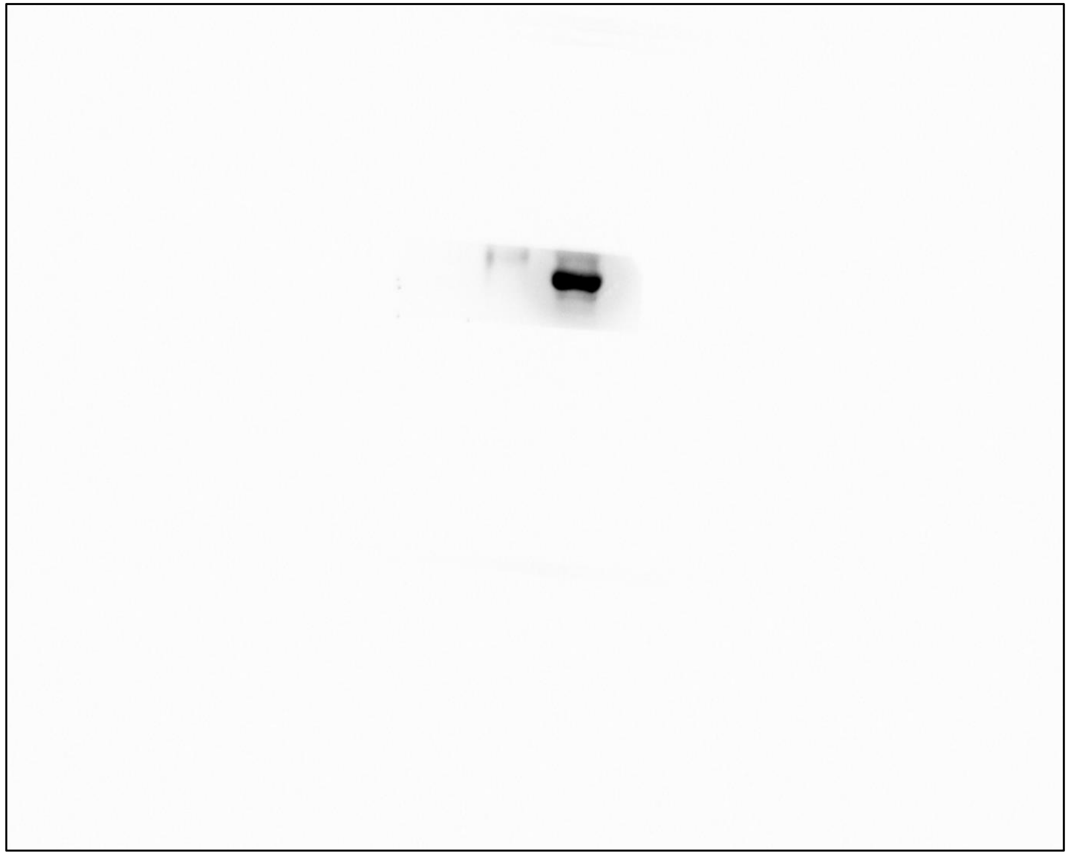

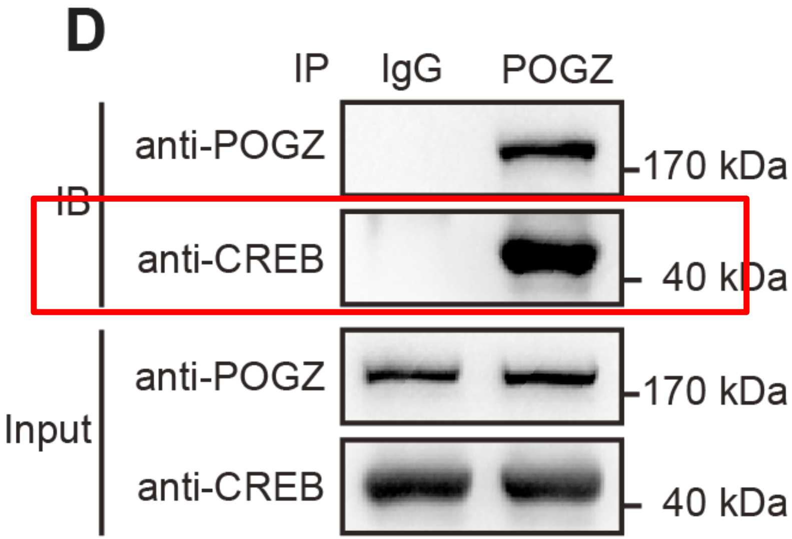

Figure 5D

Anti-POGZ

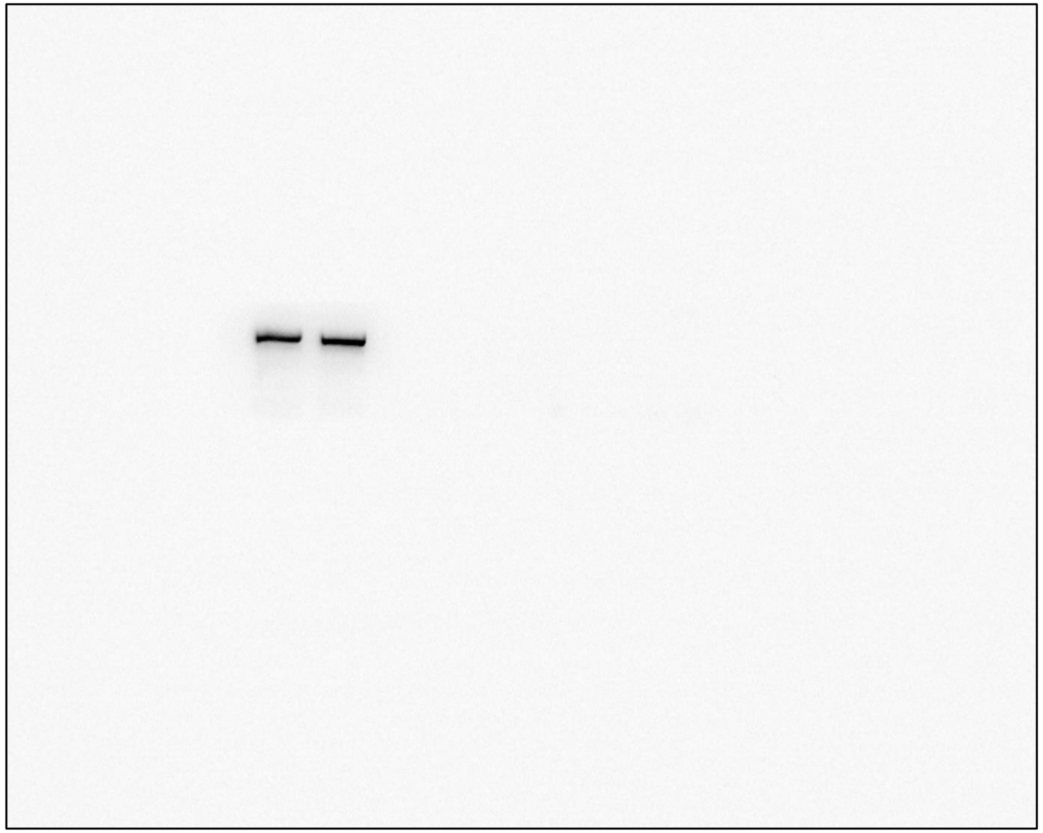

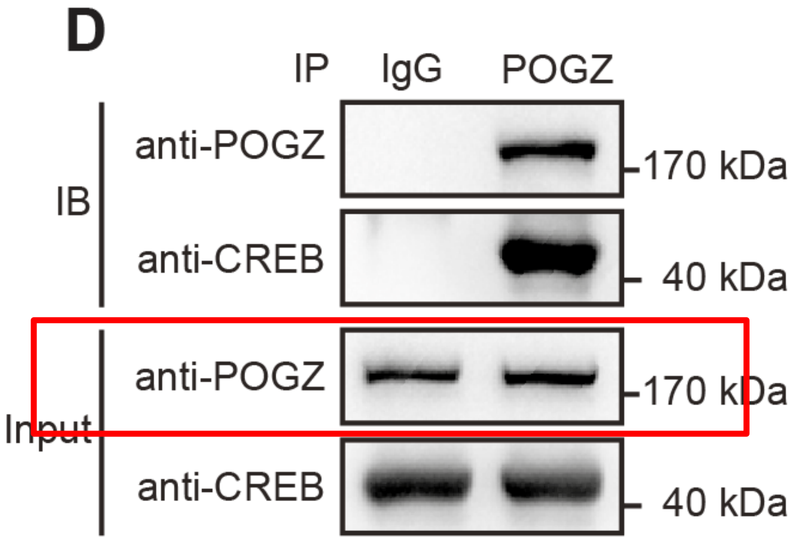

Figure 5D

Anti-CREB

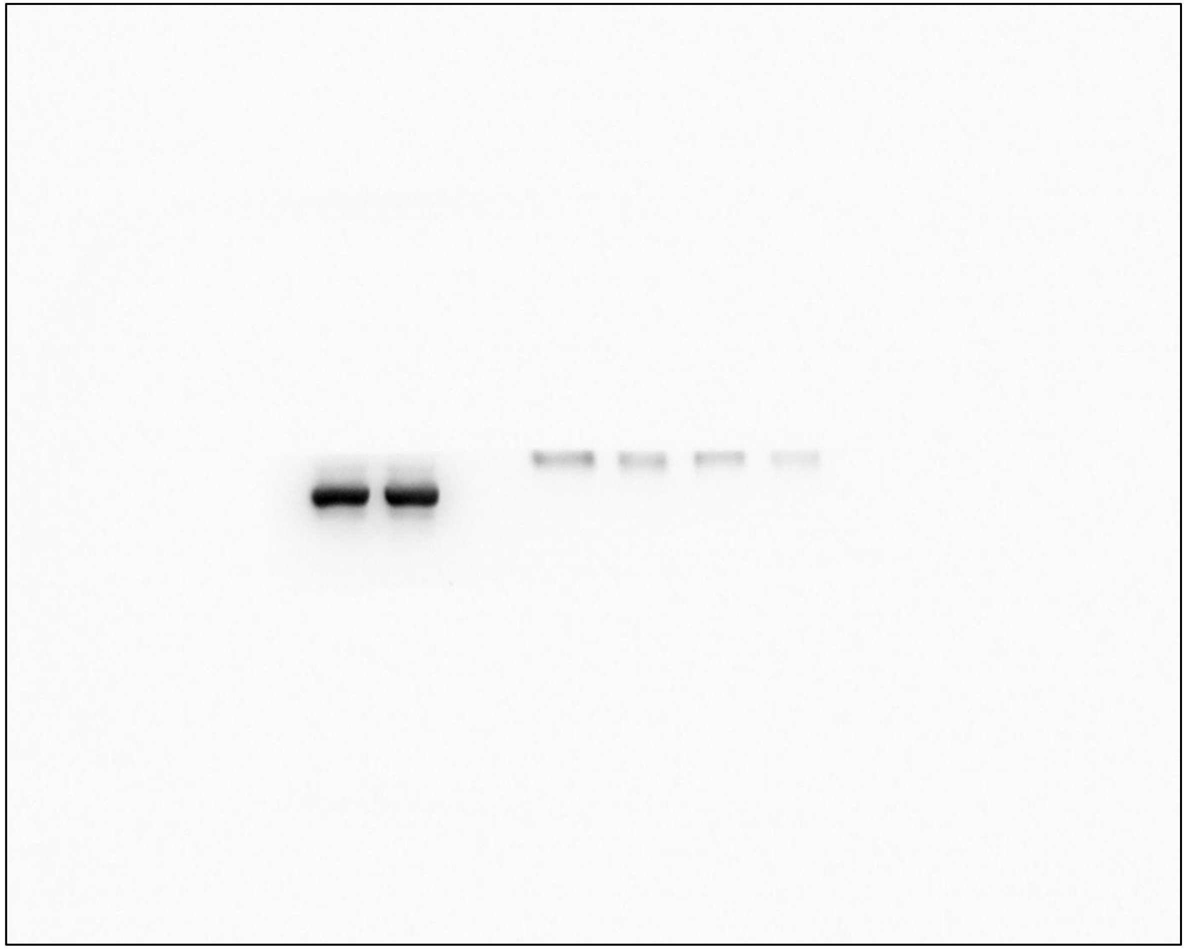

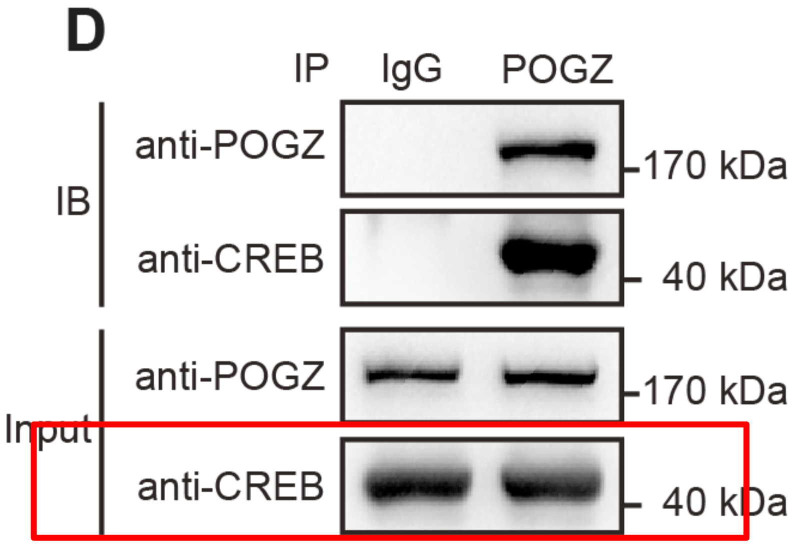

Figure 5E

Anti-POGZ

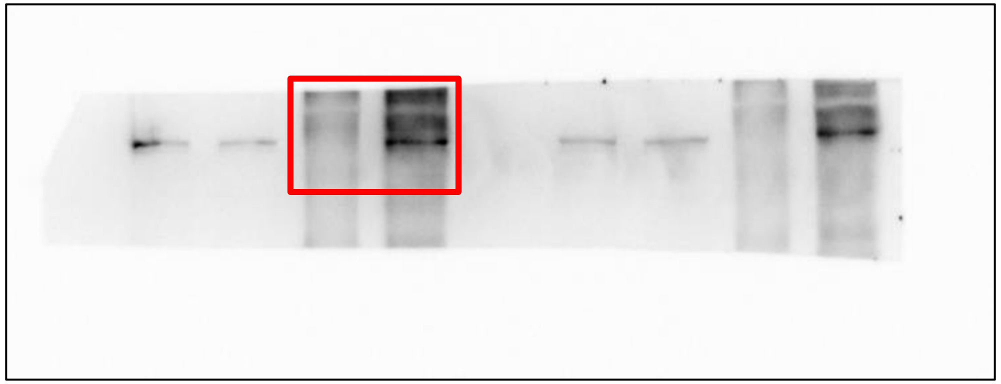

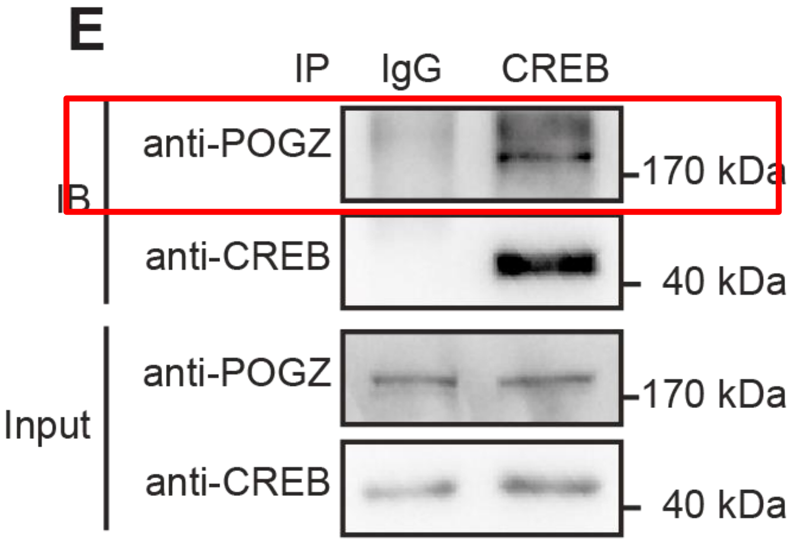

Figure 5E

Anti-CREB

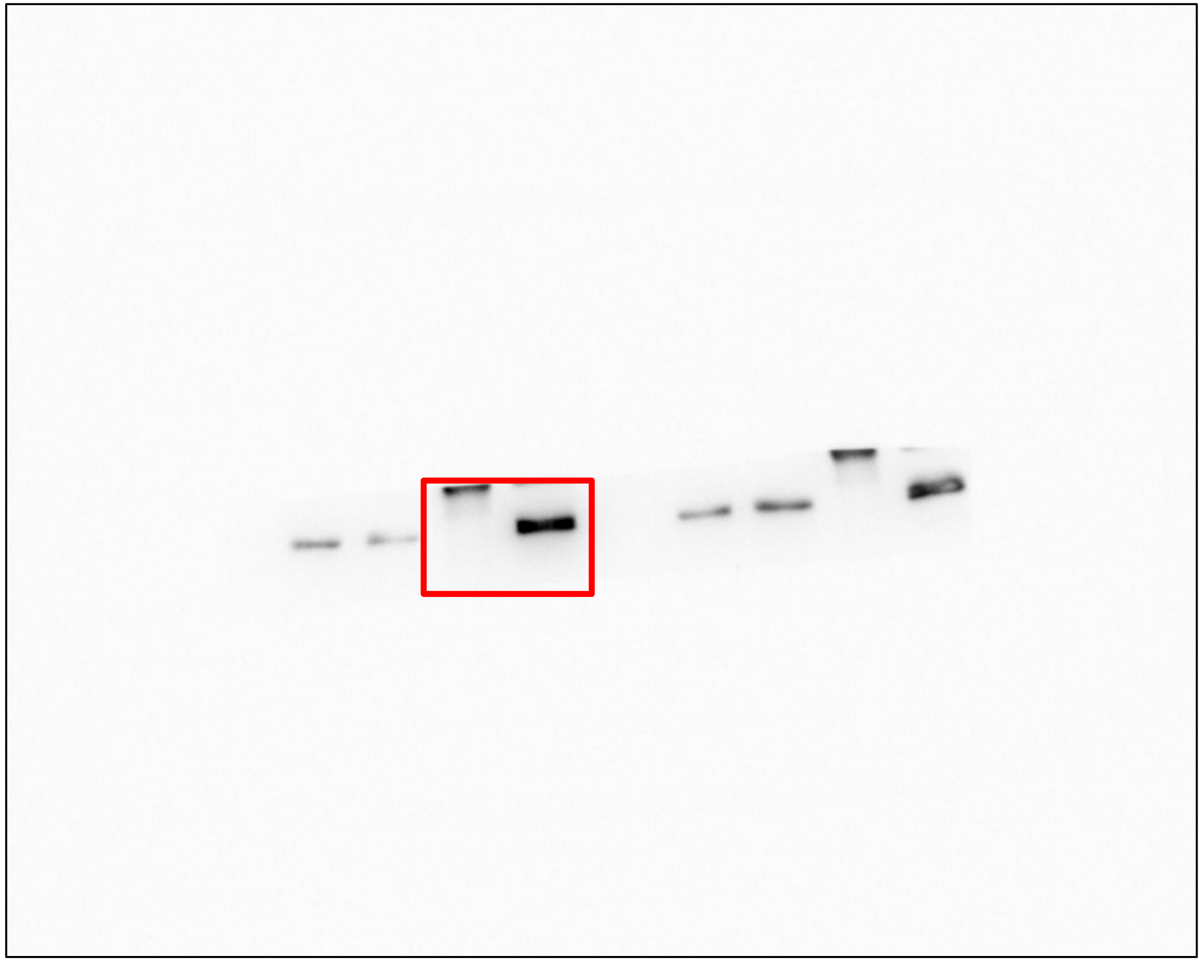

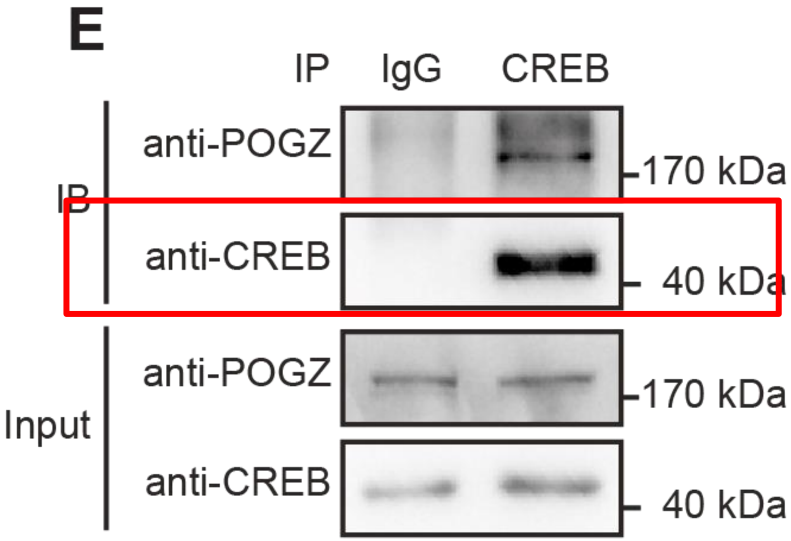

Figure 5E

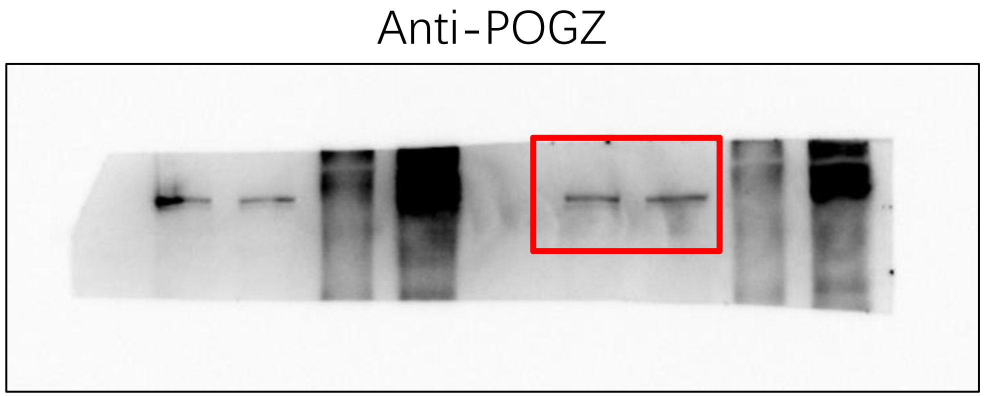
Anti-POGZ

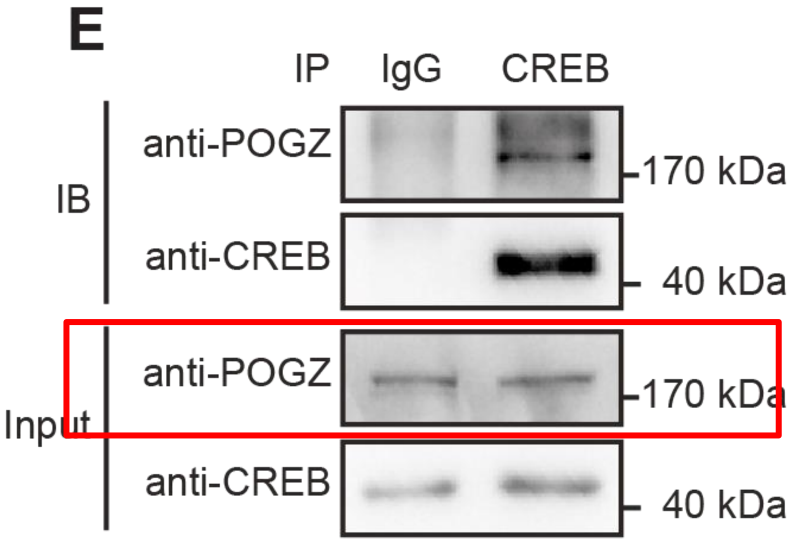
E
IP
IgG
CREB
IB
anti-POGZ
170 kDa
anti-CREB
40 kDa
Input
anti-POGZ
170 kDa
anti-CREB
40 kDa

Figure 5E

Anti-CREB

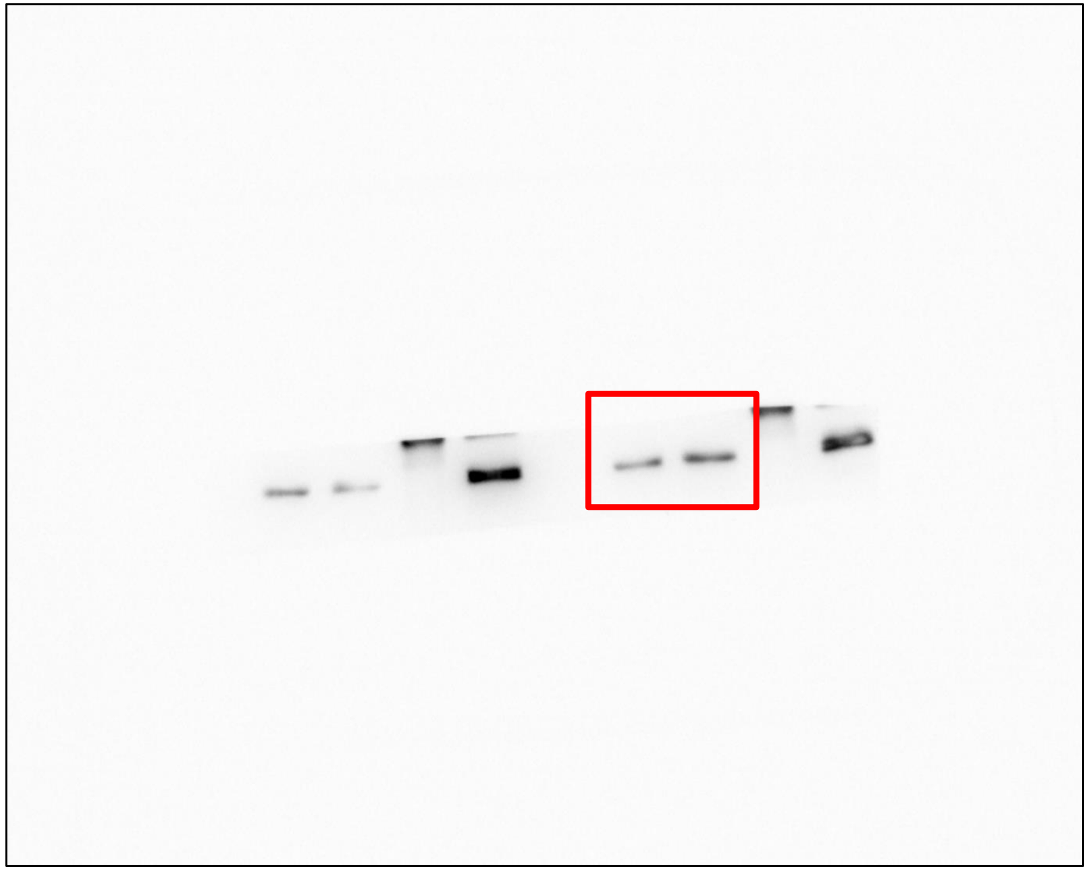

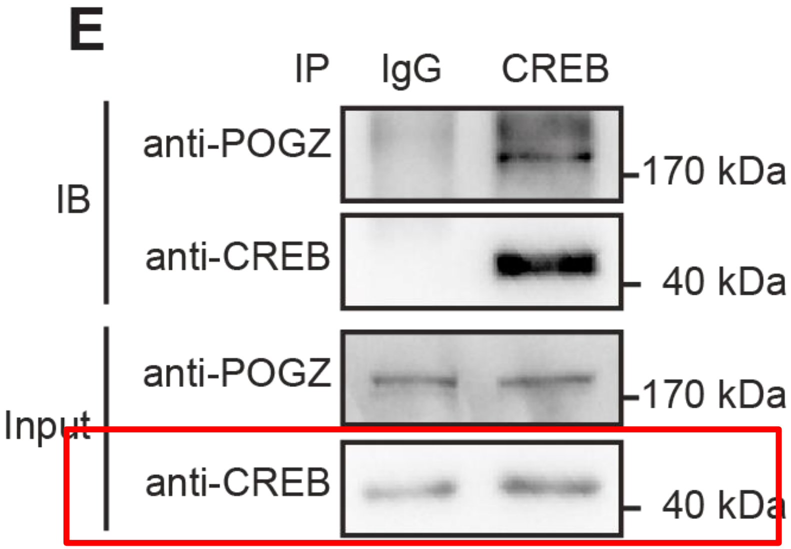

Supplement: Unedited blot and gel images [file jciinsight-11-193622-s035.pdf]
